# Review of Machine Learning for Single-Particle Tracking: Methods, Challenges, and Biophysical Insights

**DOI:** 10.1021/cbmi.5c00146

**Published:** 2025-12-01

**Authors:** Chen Zhang, Ran Liu, Zichen Ding, Peng Lu, Weiming Tian, Yan Zhao, Jiaye He, Shangguo Hou

**Affiliations:** † Institute of Systems and Physical Biology, 551667 Shenzhen Bay Laboratory, Shenzhen 518132, China; ‡ Department of Computer Science, Duke University, Durham, North Carolina 27705, United States; § School of Life Science and Technology, 47822 Harbin Institute of Technology, Harbin 150001, China; ∥ School of Electronics and Information Engineering, Harbin Institute of Technology, Harbin 150001, China; ⊥ Institute of Biomedical Health Technology and Engineering, Shenzhen Bay Laboratory, Shenzhen 518132, China; # National Innovation Center for Advanced Medical Devices, Shenzhen 518131, China; ∇ Shenzhen Institute of Advanced Technology, Chinese Academy of Sciences, Shenzhen 518055, China; ○ Shenzhen University of Advanced Technology, Shenzhen 518107, China

**Keywords:** single-particle tracking, machine learning, deep learning, particle detection, motion classification, noise reduction, biophysical inference, uncertainty quantification

## Abstract

Single-particle tracking (SPT) provides a powerful approach for probing dynamic molecular processes in living cells with high spatial and temporal resolution. Yet traditional analysis pipelines, which often rely on manual tuning or simplified models, are limited by the complexity, noise, and heterogeneity inherent to biological systems. Recent advances in machine learning (ML), especially deep learning (DL), have reshaped the SPT workflow, including particle detection, trajectory linking, motion classification, denoising, and biophysical inference. In this review, we systematically assess how ML/DL methods, including convolutional neural networks (CNNs), recurrent architectures, and Bayesian deep learning, improve the accuracy, robustness, and interpretability of SPT analyses. We survey techniques ranging from CNN-based detection and linking to statistically principled frameworks for uncertainty quantification, highlighting the versatility and effectiveness of ML/DL in overcoming persistent challenges and revealing new biological insights. We also discuss practical considerations for deployment, including selection of suitable problem domains and construction of large, high-quality training data sets. This review aims to provide a comprehensive and accessible guide to the current landscape of ML in SPT, offering both a critical evaluation of existing state-of-the-art methods and a reference for future development.

## Graphical abstract

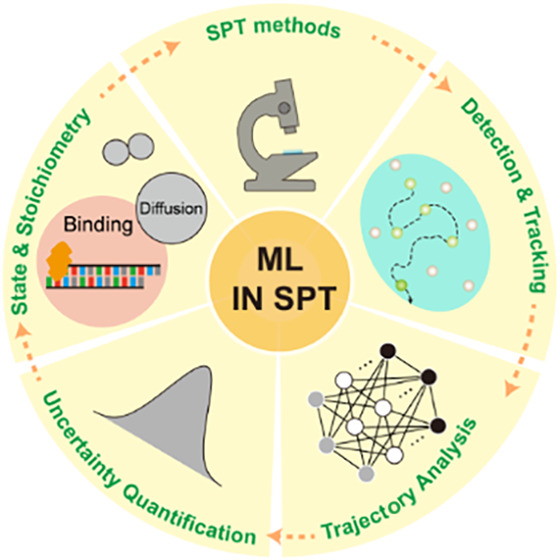

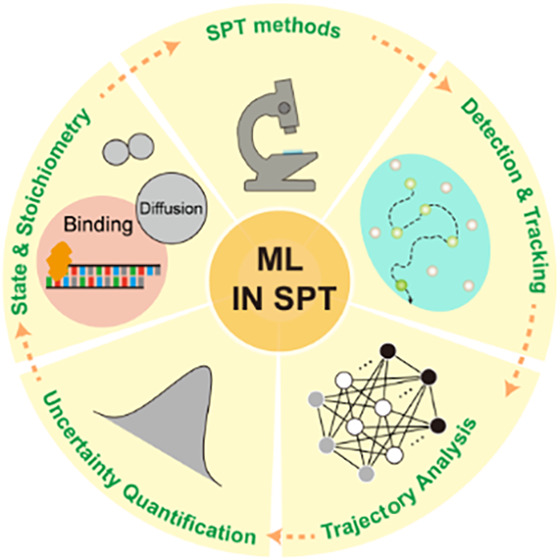

## 1 Introduction

Elucidating the behavior of biomolecules within physiologically relevant environments remains a central challenge in biophysics and cell biology.^1–12^ Optical microscopy offers a precise, noninvasive approach for visualizing biomolecular dynamic processes in situ.^10,13–21^ Among various optical imaging techniques, single-particle tracking (SPT) has emerged as a powerful method for monitoring the trajectories of individual molecules or particles in living cells and other complex biological systems.^3,9,16,22–41^ By recording molecular trajectories over time, SPT enables the detailed characterization of dynamic behaviors such as diffusion, active transport, molecular binding, and spatial compartmentalization.

However, extracting quantitative biological insights from these inherently noisy and high-dimensional data sets remains a significant challenge.^42^ Traditional analytical approaches such as mean square displacement analysis (MSD) fitting or hidden Markov models (HMMs) distill trajectories into simplified parameters and rely on a priori assumptions about state number and transition structure.^43,44^ While these strategies have provided valuable insights, their reliance on fixed models and parameters limits generalizability, motivating the adoption of more flexible ML-based frameworks.

In recent years, machine learning (ML), and in particular deep learning (DL), has been increasingly applied to address the analytical challenges associated with SPT.^42,43,45–60^ The power of ML in biological applications lies in its capacity to autonomously extract meaningful feature representations from high-dimensional, noisy data sets with minimal human intervention. By capturing subtle patterns and complex nonlinear relationships that might be inaccessible to manual analysis, ML models can significantly enhance task-specific performance in areas such as motion classification, particle trajectory linking, and signal denoising. A wide range of ML/DL frameworks, from classical algorithms like random forests to advanced deep neural networks, have been rapidly adopted by the SPT community to address the core challenges. These applications include particle detection, trajectory reconstruction, diffusion mode classification, segmentation of motion states, high-precision localization in two or three dimensions, noise suppression, and uncertainty quantification to improve analytical reliability.^42,47,48,50–58^ Notably, these data-driven approaches have demonstrated marked improvements in sensitivity, accuracy, and computational efficiency over traditional techniques in many experimental contexts.

This review summarizes recent advances in the application of ML and DL to SPT, emphasizing how various models have been employed to enhance different stages of the SPT workflow. Section 2 provides a brief overview of commonly used single-particle tracking methods. Section 3 examines ML-based approaches for particle detection and tracking (data association), highlighting how deep neural networks can effectively link particle positions across frames, particularly under crowded or high-noise conditions. Section 4 discusses trajectory analysis and classification, focusing on methods for identifying diffusive states or motion modes (e.g., distinguishing free Brownian motion from anomalous subdiffusion) and segmenting trajectories into distinct behavioral phases. Section 5 explores strategies for evaluating uncertainty in deep learning-based models. Section 6 discusses recent efforts to use ML to infer molecular states and stoichiometries from particle trajectories. Finally, we examine current limitations and the challenges of existing approaches, including issues related to model generalizability, interpretability, uncertainty quantification, and the gap between data-driven algorithms and theoretical models. We also outline opportunities in the field, such as real-time SPT analysis, integration of multimodal data, and the development of physics-informed ML frameworks.

It is worth noting that several excellent reviews have explored the application of ML in super-resolution imaging, single-molecule imaging, and trajectory analysis.^61,62^ However, to the best of our knowledge, there has not yet been a dedicated review that comprehensively addresses the application of ML in SPT, encompassing aspects such as data acquisition, data analysis, evaluation, and extraction of biophysical insights. This review highlights the recent progress in applying ML to SPT, critically evaluated its current limitations, and proposed directions for future development. By surveying the state of the art, we seek to promote further interdisciplinary innovation at the intersection of machine learning, chemical imaging, and single-molecule biophysics.

## 2 Methods of Single-Particle Tracking

Various single-particle tracking methods have been developed to investigate the molecular dynamics in living cells with an unprecedented spatial and temporal resolution. Based on the observation dimensions, these methods can be generally categorized into two-dimensional single-particle tracking (2D-SPT) and three-dimensional single-particle tracking (3D-SPT).

### 2.1 2D-SPT

2D-SPT is a widely employed approach due to its relatively straightforward implementation and capability to deliver high spatial localization precision and temporal resolution in molecular dynamics studies. Common microscopy techniques for 2D-SPT include epifluorescence microscopy (EFM), total internal reflection fluorescence microscopy (TIRFM), highly inclined and laminated optical sheet (HILO) microscopy, and light sheet fluorescence microscopy (LSFM). Each of these techniques has distinct characteristics, making them suitable for specific biological contexts (Figure 1c).

**Figure 1:**
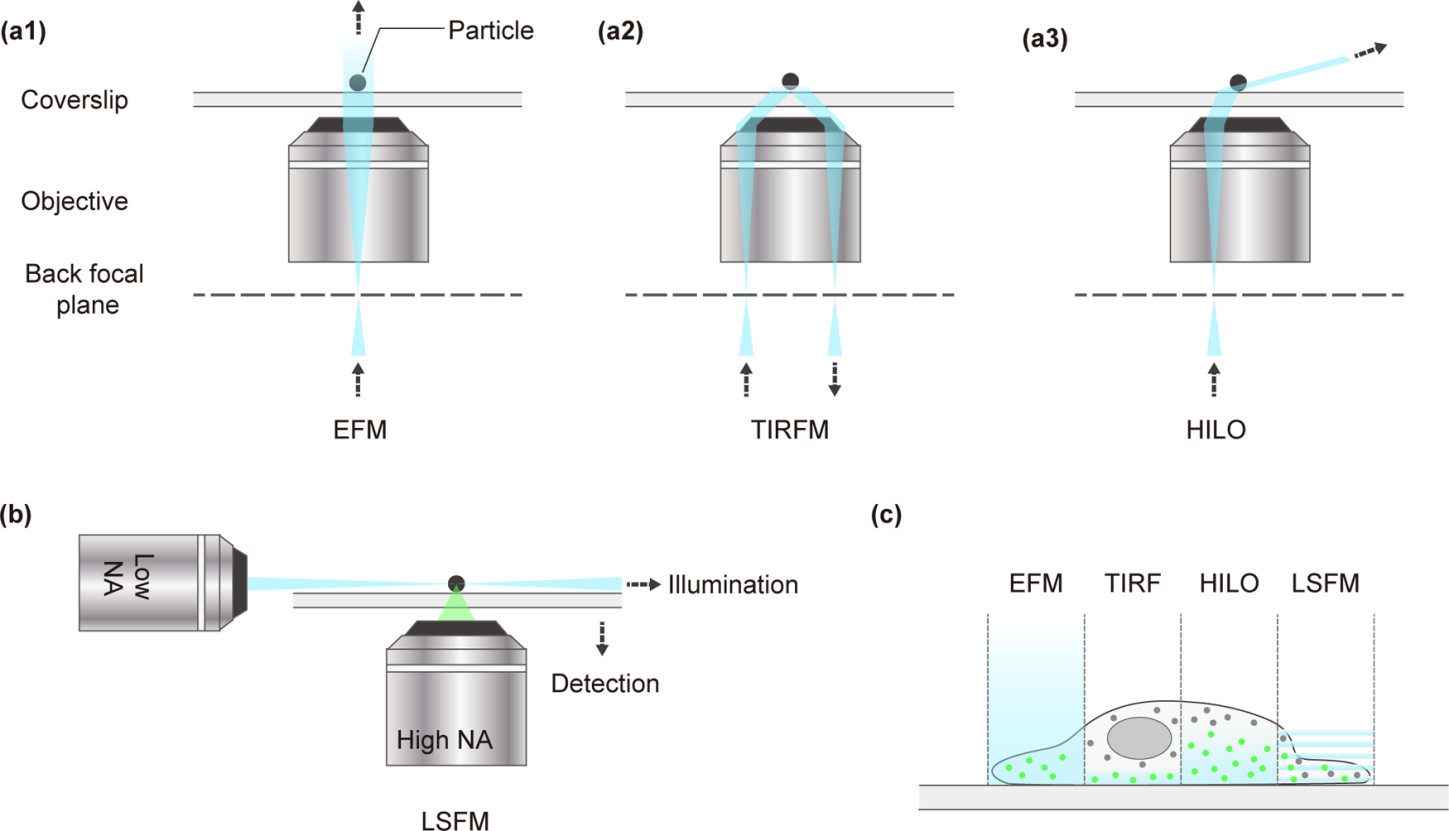
Two-dimensional single-particle tracking and imaging techniques. (a) Schematic comparison of wide-field illumination strategies commonly used in SPT, including EFM (a1), which uniformly excites the sample but generates high background; TIRFM (a2), which confines excitation to a shallow evanescent field near the coverslip; and HILO (a3), which provides inclined illumination for deeper imaging with reduced background. (b) LSFM employs a high NA detection objective or Bessel beam light illumination to perform single-molecule tracking. (c) Conceptual comparison of the illumination profiles for EFM, TIRFM, HILO, and LSFM in a cellular context. Abbreviations: NA, numerical aperture.

EFM is the most convenient technique to perform 2D-SPT. It provides wide-field illumination where excitation light passes through a filter set and dichroic mirror, illuminating the entire field of view (Figure 1a1). This approach allows straightforward imaging of fluorescently labeled molecules, which is characterized by simple implementation and relatively low cost. Building on these advantages, Cosetta et al. proposed a cost-effective assay for monitoring and characterizing the membrane receptor dynamics using EFM.^63^ However, EFM generally suffers from a relatively low signal-to-noise ratio (SNR) due to its large excitation volume, which can limit the single-molecule localization accuracy.

To overcome the low SNR of EFM, TIRFM was developed by creating an evanescent excitation field at the interface between media with differing refractive indices (Figure 1a2). TIRFM selectively excites fluorophores within approximately 100 nm of the substrate, thus substantially reducing the background fluorescence and detection volume. Consequently, TIRFM achieves spatial localization precision on the order of tens of nanometers and temporal resolution on the order of milliseconds. Although TIRFM has been successfully applied to studies of intracellular transport, as studies of flagellar internal transport reported by Engel et al.^64^ and high-precision visualization of GFP-labeled proteins in cilia reported by Wren et al.,^65^ TIRFM remains inherently constrained to the imaging depth, which only enables imaging the region in close proximity to the slide surface.

HILO microscopy circumvents the axial limitations of TIRFM by employing a slightly tilted excitation beam positioned just below the critical angle (Figure 1a3), generating a thin excitation sheet that penetrates deeper (∼10 μm) into the sample. This technique provides improved SNR compared to EFM and maintains robust spatial resolution for single-molecule tracking within thicker cellular regions. HILO has also been used to track nuclear transport dynamics in live cells, demonstrating its applicability for deeper, high-resolution imaging.^66^

LSFM further enhances the imaging depth and contrast by illuminating the sample with a laterally oriented optical sheet (Figure 1b), distinctly separating the excitation and detection paths. This method significantly reduces photobleaching and phototoxicity, provides excellent optical sectioning, and maintains high temporal and spatial resolution suitable for tracking dynamic biological processes.^67–69^

In summary, 2D-SPT using these various fluorescence microscopy methods provides robust localization accuracy and multiplex tracking capabilities (Figure 1c). However, inherent limitations remain. In particular, the inability to detect axial movements (*z*-direction) potentially leads to misinterpretation of three-dimensional particle dynamics as confined lateral movement or reduced diffusion rates. These limitations highlight the need to develop microscopy techniques aligned with specific experimental goals and biological contexts.

### 2.2 3D-SPT

Building upon 2D-SPT, 3D-SPT extends beyond the limitations of 2D techniques by enabling quantitative analysis of molecular movements in all three spatial dimensions (*x*, *y*, and *z*). This dimensional enhancement is critical for accurately capturing the heterogeneous dynamics of biomolecules within cellular environments. 3D-SPT can be broadly classified into image-based and closed-loop feedback methods.

#### 2.2.1 Image-Based 3D-SPT

A conventional approach to obtain axial information in image-based 3D single-molecule tracking is to acquire sequential 2D image stacks and link particle positions frame by frame to reconstruct 3D trajectories (Figure 2a).^70^ Although conceptually straightforward, this approach compromises temporal resolution due to sequential image collection. To overcome this limitation, point spread function (PSF) engineering techniques are employed. For example, astigmatism-based imaging employs a cylindrical lens to introduce controlled optical aberration, resulting in asymmetric PSFs whose shape varies with axial displacement.^71^ In the absence of a cylindrical lens, the PSF remains symmetrical and circular at the focal plane. Upon insertion of the cylindrical lens, the beam experiences unequal convergence along the two orthogonal axes, causing the PSF to appear as ellipses of different orientations and eccentricities above and below the focal plane. This shape variation enables nanometer-scale inference of the emitter’s axial position, thereby achieving high-precision 3D localization (Figure 2b). More advanced PSF engineering, such as double-helix PSF (DH-PSF), employs spatial light modulators (SLMs) or phase masks to generate a characteristic PSF consisting of two symmetric lobes with identical size, intensity, and shape. The azimuthal angle of the line connecting these two lobes rotates with axial defocus, and the rotation angle increases linearly with the degree of defocus, thereby enabling high-precision three-dimensional localization (Figure 2c).^72,73^ This method offers high localization precision (∼25 nm in *x*–*y* and ∼50 nm in *z* dimensions), as demonstrated in the tracking of single mRNA particles within yeast cells.^74^ While this approach substantially extends the axial range of accurate particle tracking, it necessitates the use of customized high-brightness fluorophores and precise calibration of the phase mask or SLMs, thereby increasing the complexity and alignment sensitivity of the optical system. Other engineered PSFs, such as tetrapod PSF^75^ and vortex PSF,^76^ have also been developed to optimize depth sensitivity and resolution. While these approaches extend the axial tracking range significantly, they require high-brightness fluorophores and precise calibration of optical components, increasing system complexity.

**Figure 2:**
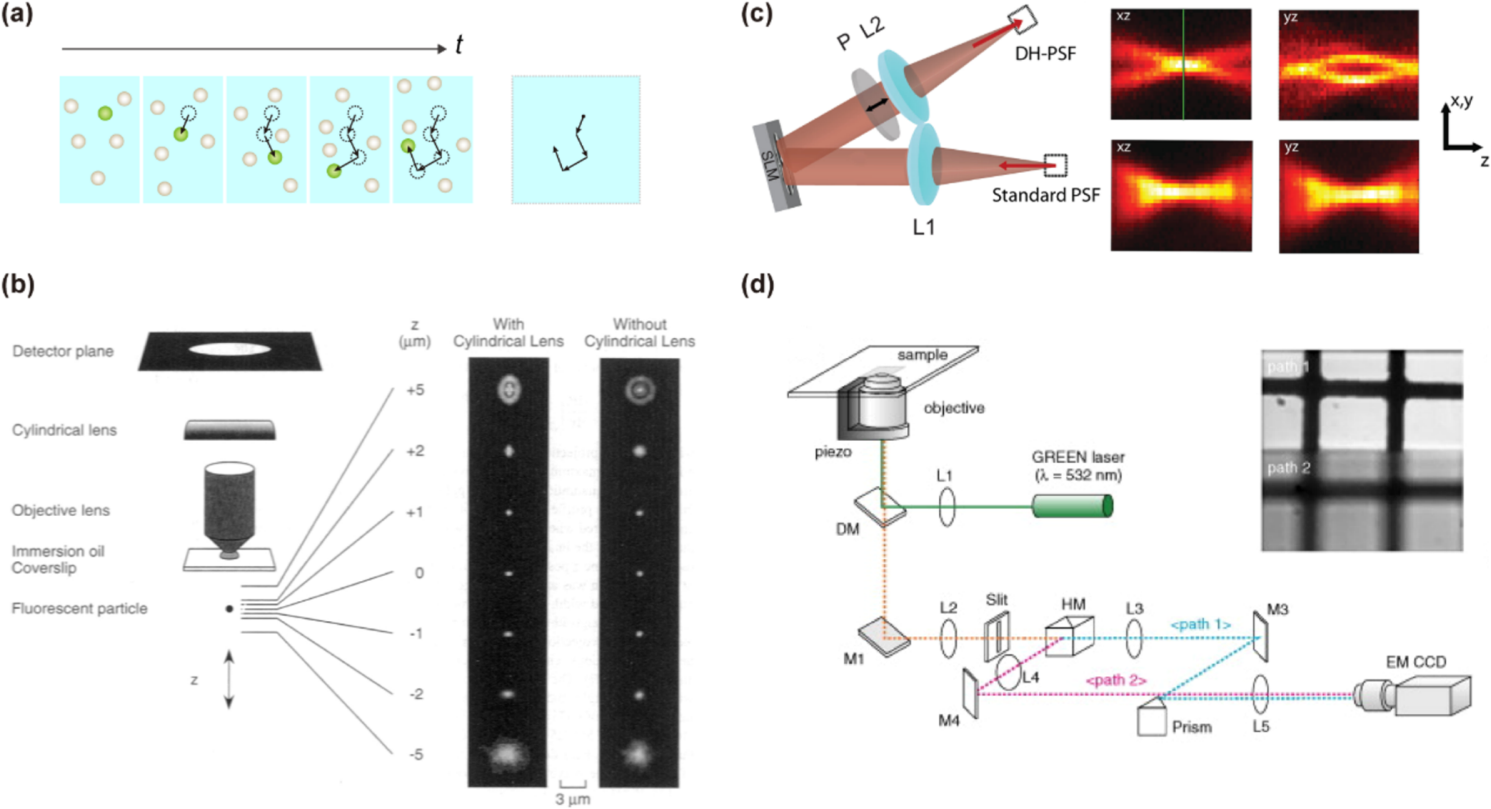
Image-based three-dimensional single-particle tracking and imaging techniques. **(**a) Image-based tracking.^70^ Axial information is obtained by stacking a series of 2D images to generate 3D images and linking the particle positions across frames. (b) PSF engineering based on astigmatism, where astigmatism is introduced by placing a weak cylindrical lens in the detection path, generating asymmetric PSFs whose shape variations enable accurate determination of the emitter’s axial position. Adapted with permission from ref 71. Copyright 1994 Elsevier. (c) In PSF engineering, an SLM or phase mask placed in a 4f optical system modulates the emitted light phase to produce distinct PSF patterns (e.g., dual rotating lobes) that change with the emitter’s axial position, enabling accurate 3D localization. Adapted with permission from ref 73. Copyright 2010 American Chemical Society. (d) Dual-plane imaging using an EM-CCD camera. Two focal planes are recorded simultaneously, allowing axial localization from interplane intensity or positional differences without the need for engineered PSFs. Adapted with permission from ref 77. Copyright 2007 Elsevier. Abbreviations: t, time of shooting; P, polarizer; L, lens; DM, dichroic mirror; M, mirror; and HM, half mirror cube.

Another image-based method is biplane imaging, where the fluorescence is split into two channels by a spectroscope or prism and is imaged on two planes with known focal lengths. When the particle moves in the *z*-direction, the relative position and intensity ratio of the image points on the two planes change, and the Z coordinate can be calculated according to the changes, without special PSF design. (Figure 2d). Relative intensity or positional differences between the two images provide a robust basis for *z*-position retrieval. One of the earliest demonstrations was by Watanabe et al.,^77^ who developed a bifocal imaging system using a beam splitter and a single CCD camera to simultaneously capture a focused and defocused image of the same particle. This setup enabled 3D tracking of fluorescent beads and melanosomes in live cells with high spatial precision at millisecond time scales under favorable signal-to-noise conditions. Building upon this, Park et al.^78^ applied a dual-plane imaging approach to track individual quantum dot–labeled synaptic vesicles in live hippocampal neurons, achieving ∼8.5 nm lateral and ∼12 nm axial resolution at video-rate acquisition, and revealing vesicle behavior leading up to neurotransmitter release. Extensions like multifocal plane microscopy (MPM), which record multiple focal planes simultaneously, further enhance axial resolution and tracking continuity.^79^

Although these methods enable axial position determination within a single camera exposure, their temporal resolution remains constrained by the camera’s readout speed. Additionally, the axial tracking range is often limited, typically requiring complex optical designs or a trade-off in the photon throughput to extend the depth coverage.

#### 2.2.2 Closed-Loop Feedback 3D-SPT

Closed-loop feedback 3D-SPT methods dynamically maintain the particle within the imaging volume by real-time adjustment, thereby improving the axial tracking range and temporal resolution. These methods can be categorized into patterned excitation and modified detection approaches, each offering distinct advantages for real-time molecular tracking.

##### 2.2.2.1 Patterned Excitation Methods

In the patterned excitation method, real-time tracking is achieved by modulating the excitation beam position or shape to probe spatial locations (Figure 3a). Tetrahedral excitation-based 3D tracking employs four laser beams arranged in a tetrahedral geometry around the focal volume.^31,32^ Axial and lateral displacements of molecules from the center are inferred from intensity variations across these beams. A feedback system adjusts the position of a piezo stage in real time, keeping the excitation volume centered around the particle. While tetrahedral excitation relies on multibeam intensity comparison, orbital scanning tracking achieves 3D localization by circularly scanning a laser beam around the particle (Figure 3b).^29^ Deviations of the particle from the center produce sinusoidal fluorescence intensity changes. Axial position determination is enhanced by modulating the scan height (helical scanning), allowing for precise estimation of axial movements. Feedback mechanisms continually reposition the laser scan to track the particle. Knight’s Tour scanning, on the other hand, employs a discontinuous, chess knight-like pattern where the excitation beam sequentially moves between discrete points around the particle (Figure 3c).^34,37,38^ This method efficiently covers the tracking area, reducing time delays and phototoxicity and improving localization speed and accuracy. Collectively, these patterned excitation strategies achieve high-precision 3D localization by coupling beam modulation with fluorescence feedback, each offering unique trade-offs in spatial resolution, temporal response, and implementation complexity.

**Figure 3:**
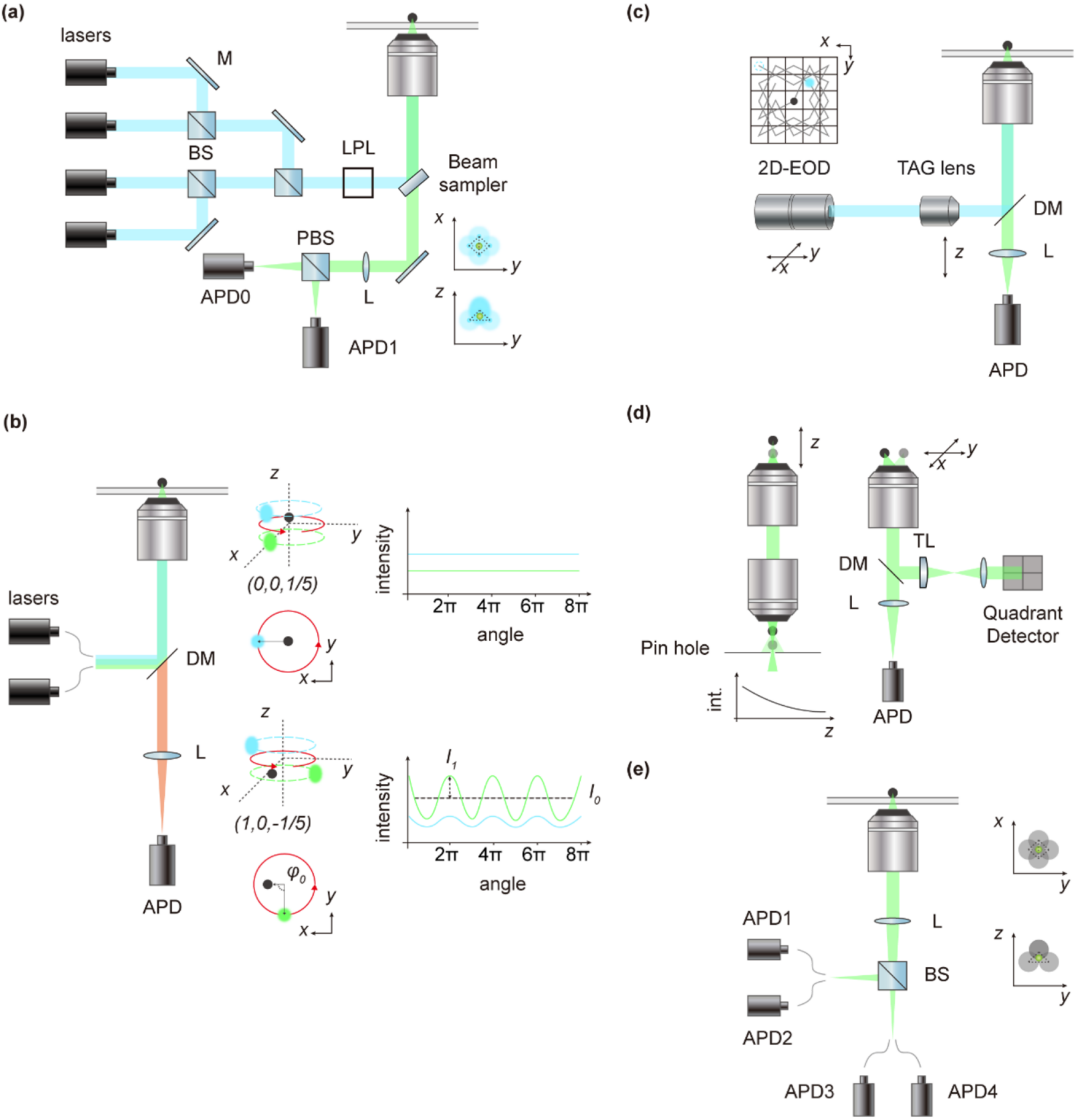
Feedback-based three-dimensional single-particle tracking and imaging techniques. (a) Tetrahedral excitation-based tracking.^31^ Particle displacement relative to the tetrahedral excitation center is inferred from photon count asymmetry among four beams, enabling real-time 3D localization and feedback. (b) Orbital Tracking.^29^ A circular laser scan induces fluorescence modulation upon particle displacement; phase (φ_0_) and amplitude (I_1_) of the signal reveal direction and radial offset from the orbit center. (c) 3D position acquisition via rapid tracking (3D-PART).^37^ A two-dimensional electro-optical deflector (2D-EOD) executes a lateral knight’s tour scan, combined with axial scanning by a tunable acoustic gradient (TAG) lens, forming a three-dimensional scanning volume. (d) Split detection-based 3D tracking.^24^ Lateral positions are resolved via quadrant detector signals, while the axial position information is obtained from the fluorescence gradients detected postpinhole. (e) Tetrahedral detection-based tracking. Four single-photon counting avalanche photodiodes (APD) arranged tetrahedrally detect fluorescence asymmetry, with two detectors resolving lateral displacement and the other two enabling axial localization. Abbreviations: BS, beam splitter; LPL, linear polarizer; and TL, tube lens.

##### 2.2.2.2 Modified Detection Methods

In contrast, modified detection analyzes the spatial distribution of fluorescence signals to extract the positions of molecules. For example, split detection-based 3D tracking involves spatially separating the fluorescence signal into multiple detection channels (Figure 3d),^24^ where photon count differences across channels precisely encode the particle’s 3D position, enabling real-time feedback control. Building on this principle, tetrahedral detection-based tracking utilizes four detectors (such as fiber-coupled single-photon avalanche diodes, SPADs) arranged in a tetrahedral geometry to enhance axial sensitivity and isotropic localization precision. (Figure 3e).^25,27^ By comparison of fluorescence photon counts simultaneously collected from different spatial directions, this approach rapidly infers particle displacement and provides immediate feedback to reposition the focal volume for continuous 3D tracking.

In summary, 3D-SPT methods significantly extend the spatial and temporal resolution of particle tracking, thereby overcoming the inherent limitations of 2D tracking and providing comprehensive insights into complex cellular processes across all spatial dimensions.

## 3 Machine Learning for Particle Detection and Tracking in SPT

Single-particle tracking experiments produce long sequences of microscopy images in which tiny, diffraction-limited spots must be accurately identified and tracked over time. Traditional detection methods (e.g., intensity thresholding and centroid finding) and tracking (e.g., frame-to-frame linking by nearest-neighbor or Kalman filtering) often struggle in the noisy, crowded, and low-contrast environments typical of live-cell imaging.^80^ These algorithms require careful parameter tuning for each data set and tend to fail under low-SNR conditions, often necessitating extensive user intervention. Machine learning offers a powerful, data-driven alternative by learning to detect and track particles directly from examples, without relying on rigid, predefined rules.^52^ In recent years, ML/DL approaches have significantly advanced the accuracy, robustness, and efficiency of particle detection and trajectory reconstruction in SPT. This section provides a conceptual overview of how these methods enhance data acquisition in SPT, from identifying particle locations in noisy images to linking those positions into trajectories and how they are deployed in practice via software and hardware implementations.

### 3.1 Enhancing Particle Detection with Deep Learning

Deep neural networks (DNNs) have become essential tools for identifying and localizing particles in microscopy images. Unlike traditional algorithms that rely on simple thresholding and centroid calculations, deep learning models can learn to recognize the visual characteristics of particles and distinguish them from noise or background directly from training data. For example, convolutional neural networks (CNNs) can be trained not only to detect the presence of particles but also to predict their precise coordinates with subpixel accuracy. One notable example is DeepTrack (Figure 4a), a CNN-based tracker that achieves nanometer-level localization across a wide range of particle types and imaging conditions.^81^ DeepTrack has been shown to significantly outperform traditional methods, such as centroid or radial-symmetry algorithms, particularly under low-SNR or uneven illumination. By learning directly from annotated images, the network automatically identifies the most effective features for particle detection, eliminating the need for manual parameter tuning and improving reliability even under difficult imaging conditions. CNNs were also used for multidimensional information detection. Another automated multidimensional SPT platform coupled to a CNN recognizes defocused image patterns of anisotropic gold nanoprobes to recover 3D orientation during tracking (Figure 4b), thereby turning a difficult pattern-matching detection problem into a learned regression task.^59^ The CNN remained accurate at low SNRs where conventional pattern-matching with correlation coefficients failed. With S/N ≈ 4 (typical for live-cell imaging), orientation errors were <2° for both azimuth and polar angles; at S/N ≈ 2, the CNN’s mean errors increased only modestly (∼3.3° and ∼2.0°), whereas the correlation method degraded severely (∼19.3° and ∼9.1°). These results directly illustrate how deep learning strengthens the “detection” step by separating weak, structured particle signals from background and by generalizing beyond the finite, noisy template libraries that limit classical approaches.

**Figure 4:**
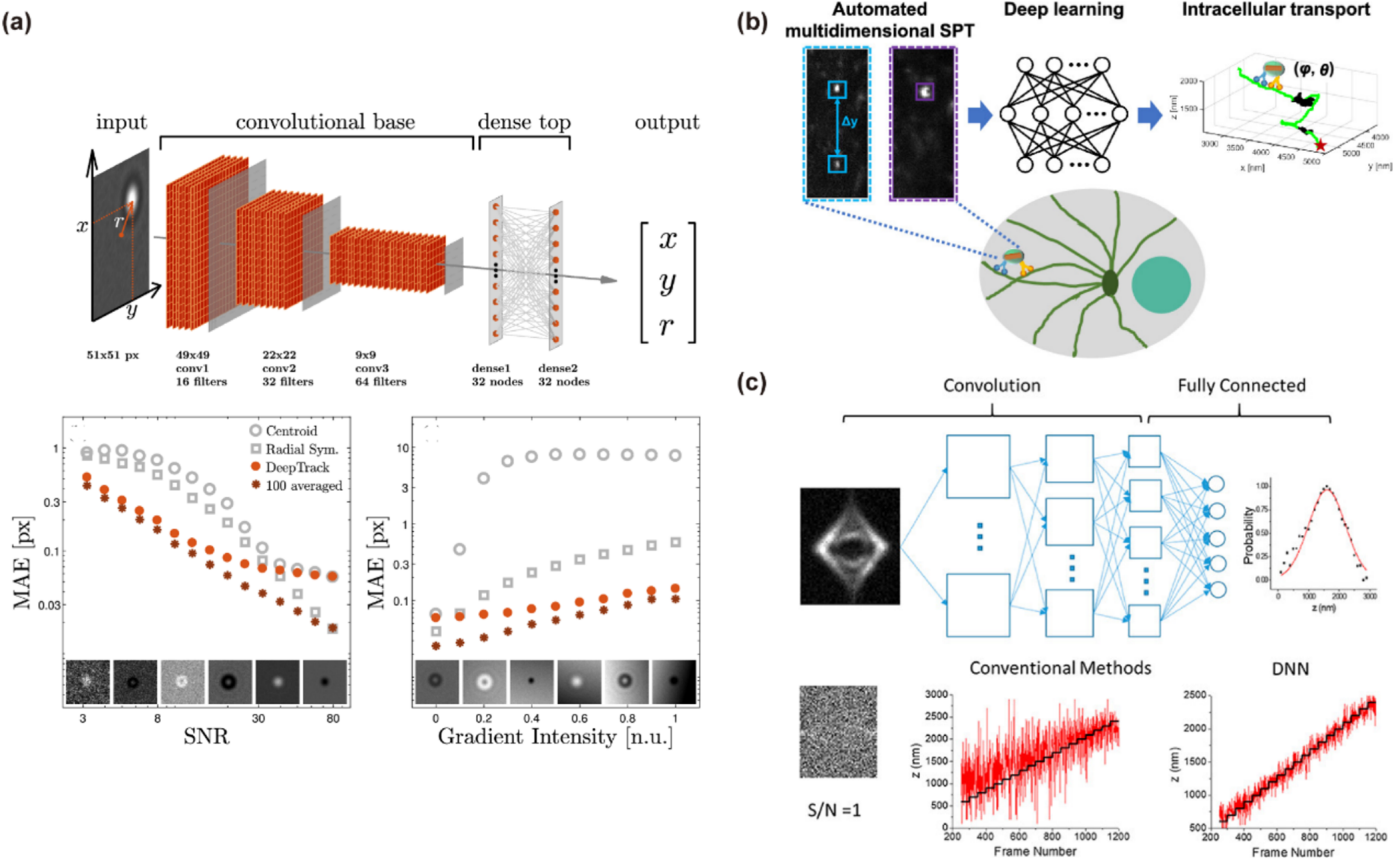
Deep neural networks for recognizing and localizing particles in microscopy images. (a) DeepTrack neural-network architecture and performance. Adapted with permission from ref 81. Copyright 2018 Optical Society of America. (b) Schematic diagram of the automated high-speed multidimensional SPT imaging setup combined with a deep learning model (CNN-sim+exp model). Adapted with permission from ref 59. Copyright 2024 American Chemical Society. (c) Noise-resistant 3D single-particle tracking based on DNNs. Adapted with permission from ref 48. Copyright 2018 American Chemical Society.

Another key advantage of deep learning is its ability to denoise and enhance weak signals. Encoder–decoder architectures, such as U-Net convolutional networks, can be trained to transform raw, noisy images into cleaned-up outputs or probability maps that highlight particles while suppressing background. This “learned filtering” greatly facilitates subsequent localization steps. Essentially, the network functions as a powerful nonlinear filter that preserves particle signals and reduces background noise, enabling the detection of dim spots that traditional methods might miss. For example, in one demonstration involving 3D particle tracking with engineered point spread functions, deep neural networks successfully recovered particle positions even at SNR as low as ∼1 (Figure 4c), where conventional cross-correlation techniques failed to detect the particles.^48^ This noise robustness enabled researchers to reduce camera exposure times to just 50 μs for high-speed imaging without sacrificing localization accuracy. Such advances highlight how machine learning-based detection pushes the boundaries of spatial and temporal resolution in single-particle tracking, extracting more information from each frame than traditional filtering methods ever could.

At the core of these methods, convolutional layers are highly effective at extracting features from images and learning to recognize the brightness and shape patterns that indicate the presence of a particle. Many workflows begin with a CNN classifier to identify candidate regions, followed by a secondary network or refinement step (e.g., coordinate regression or fine-grained heatmaps) to identify particle centers with high precision. Other approaches employ fully convolutional networks (FCNs) or U-Net architectures to generate segmentation masks that highlight particle locations across the image. The final output is a set of particle coordinates (often with associated uncertainties) for each frame, all achieved with minimal human intervention. Notably, these networks can be trained on simulated data when experimental annotations are scarce using synthetic particle images with known ground truth positions. In doing so, the network implicitly learns the characteristics of the imaging system’s point spread function and noise profile. Multiple studies have shown that such trained models consistently outperform traditional detection algorithms in both accuracy and sensitivity across diverse SPT data sets.

### 3.2 Automated Trajectory Linking and Motion Tracking

Detecting particle positions in individual frames is only part of the challenge. Another challenge is that these positions must also be accurately connected into trajectories that reflect each particle’s movement over time. This step, known as data association or linking, becomes especially difficult when particles approach one another closely, momentarily vanish, or exhibit unpredictable motion. Traditional tracking methods (such as nearest-neighbor linking or the Hungarian algorithm for minimizing gaps) depend heavily on predefined motion assumptions or user-specified thresholds (e.g., maximum displacement allowed per frame). These methods often fail when particles move quickly or erratically. Machine learning introduces innovative, data-driven solutions for trajectory linking by treating tracking as a pattern-recognition task across entire sequences rather than a simple frame-by-frame matching problem.

One promising approach uses recurrent neural networks (RNNs), such as long short-term memory (LSTM) networks, to learn the dynamics of particle motion. Rather than relying on overly simplistic models like Brownian or directed motion, an RNN can be trained on trajectory data to predict a particle’s likely position in the next frame based on its recent history. These predictions help guide the linking process by assigning a probability or “cost” that a detection in frame *t + 1* belongs to the same particle as a trajectory ending in frame *t*. For example, Yao et al. developed a deep learning-based data association method that integrates both CNNs and LSTM modules to improve tracking performance.^52^ In this framework, a CNN first analyzes small image patches around each particle to extract visual features (Figure 5), while the LSTM processes the sequence of past positions to learn the particle’s motion pattern over time. The network then assigns a score to each potential link, effectively learning the best association strategy from data rather than relying on handcrafted rules. When tested on challenging microscopy videos, this deep learning-based tracker achieved state-of-the-art performance, matching or even surpassing human experts in accurately reconstructing particle trajectories. By learning motion constraints such as typical diffusion rates or directional persistence, the model can reliably maintain particle identity even in crowded environments where simple distance-based linking would fail.

**Figure 5:**
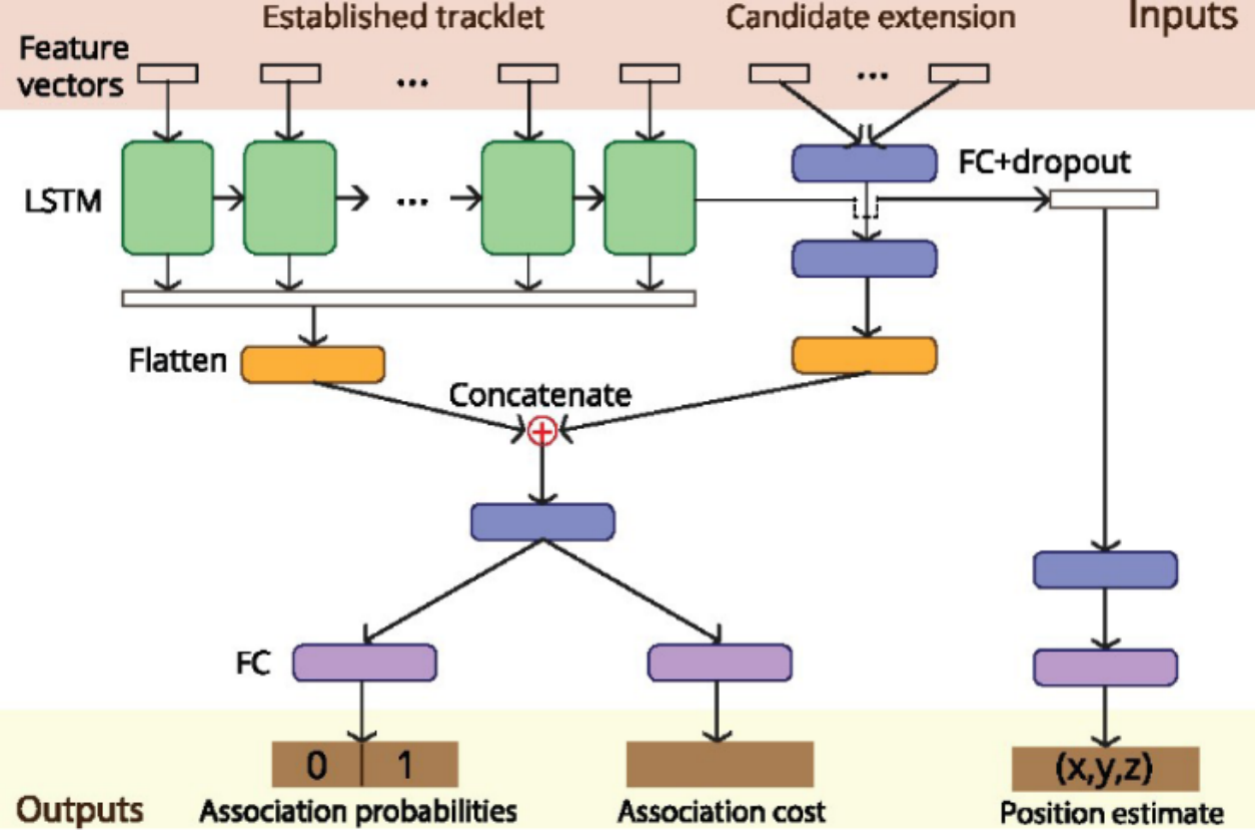
An example of DL application in data association in SPT. Yao et al. introduced a model integrating CNN and the LSTM, which evaluates motion dynamics from established tracklets and candidate extensions using a combination of learned and handcrafted features. Tracklet–candidate pairs are scored for association likelihood, and predicted positions are output via multitask learning. Adapted with permission from ref 52 Copyright 2020 Oxford University Press.

It is important to note that many current SPT analyses use hybrid approaches, for example, applying a CNN to detect particle positions, followed by a machine learning-based filter or predictor that assists a traditional tracker in linking those detections. This approach combines the transparency and interpretability of classic tracking algorithms with the adaptability of machine learning. Overall, the integration of ML and deep learning in trajectory reconstruction has significantly increased both the quantity and accuracy of tracked trajectories, especially in crowded or noisy data sets where conventional methods often fail or confuse tracks. By automating the linking process, these approaches enable high-throughput studies, allowing thousands of trajectories to be reliably extracted without manual intervention.

### 3.3 Real-Time and Hardware-Accelerated SPT Workflows

While software-based machine learning methods have brought impressive gains in accuracy, another advancement is the integration of these models into real-time or near-real-time SPT workflows through optimized hardware. Traditionally, deep learning involves training a model on a powerful graphics processing unit (GPU) server and performing inference on image batches as a postprocessing step. However, for live-cell experiments and high-throughput screening, there is increasing demand to perform particle detection and tracking instantly as images are captured. To meet the requirements for speed and low latency, researchers are adopting hardware-accelerated implementations of ML models, leveraging GPUs, tensor processing units (TPUs), and field-programmable gate arrays (FPGAs).

Modern GPUs are well-suited to handle convolutional computations of CNNs and can often process frames in real time (e.g., >30 frames per second) even when running complex models, especially if the region of interest is small or a high-end card is used. As a result, many laboratories now implement inference pipelines that stream microscope images directly to GPU-accelerated programs, enabling near-real-time particle detection and localization in each frame. Such setups make real-time feedback possible, for example, adjusting focus or illumination based on particle positions, or triggering high-resolution imaging when a particle of interest is detected.

For applications with even more demanding real-time requirements, FPGAs and custom hardware offer a powerful solution. These platforms can run neural networks with an extremely low latency. Using tools like HLS4ML, FPGAs can be programmed to implement trained neural networks directly in logic gates (Figure 6), enabling inference to be performed in just a few microseconds.^82^ For example, Barbosa et al. demonstrated that an FPGA-based ML filter could make particle identification and tracking decisions with submicrosecond latency.^83^ In their study, this allowed real-time data filtering in particle physics detectors to reduce the bandwidth, but the same concept applies to optical SPT: an FPGA placed near the camera could, in principle, detect particles in each 2D frame almost instantly and transmit only the coordinates or other distilled outputs. With the growing capabilities of FPGAs and AI accelerators, it is becoming increasingly feasible to embed advanced algorithms directly into the data acquisition pipeline, transforming analysis from a postprocessing step into an integral part of real-time imaging.

**Figure 6:**
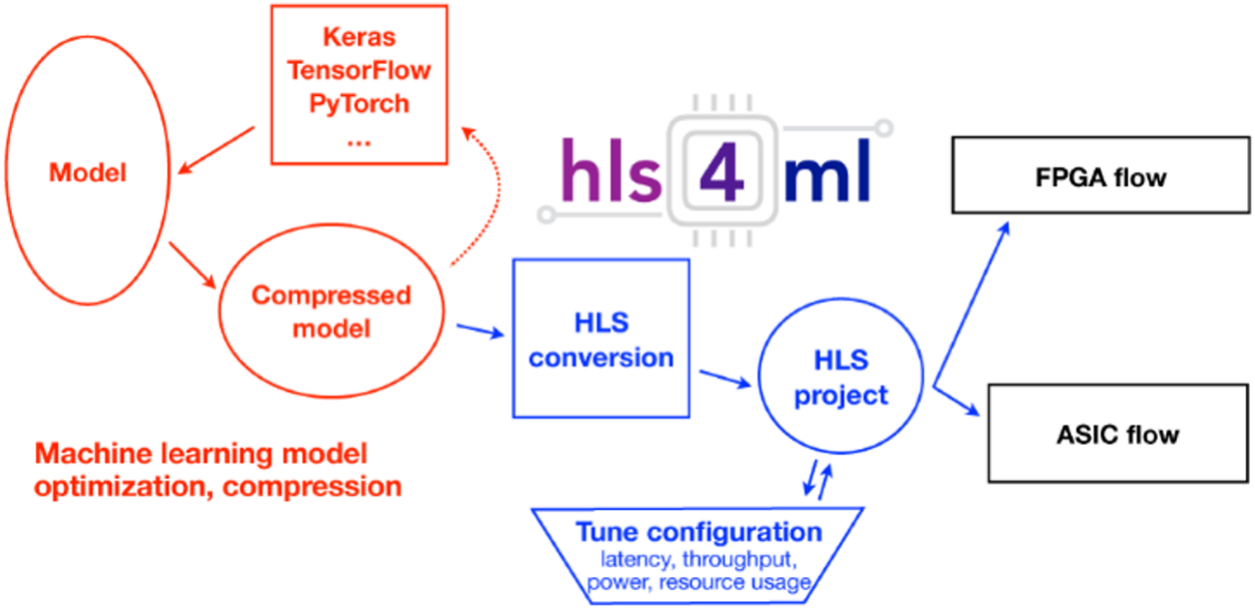
Overall workflow of hls4 mL introduced by Fahim et al., an open-source workflow including a model that translates trained ML models from frameworks like Keras and PyTorch into FPGA or ASIC implementations via high-level synthesis (HLS), integrating model compression, hardware-aware configuration tuning (e.g., latency, resource usage), and exporting into HLS-compatible projects. Adapted with permission from ref 82. Copyright 2021 arXiv.

Deploying ML models in hardware allows SPT experiments to process far larger data volumes with higher throughput. Instead of storing large-sized raw video, data can be analyzed and compressed in real time, retaining only essential trajectory information. This is crucial for high-throughput screening, where hundreds of cells or conditions are imaged in parallel, making ML-driven automation the only practical solution. Real-time tracking also enables closed-loop experiments, allowing experimental parameters to be adjusted on the fly based on particle behavior, such as following a molecule with a movable stage or triggering photobleaching when it enters a specific region.

In summary, machine learning has become a transformative force for single-particle tracking, enhancing every stage from data acquisition to analysis. ML and deep learning models, such as CNNs, RNNs, and autoencoders, have improved localization accuracy, trajectory linking, and robustness in challenging conditions like low SNR, dense fields, and complex motion, all while reducing manual effort.^48,52^ On the hardware side, GPU and FPGA implementations are driving real-time, high-throughput tracking in live-cell experiments. As both ML models and hardware continue to advance, SPT workflows become more automated, intelligent, and responsive. This progress is accelerating scientific discovery and making dynamic, real-time experimentation possible in ways that were previously unattainable.^59^

## 4 Learning from Trajectories: Classification and State Identification

The development of machine learning, particularly deep learning, has propelled trajectory analysis beyond empirical rules and predefined statistical models toward data-driven intelligent approaches. Currently, trajectory analysis can be broadly categorized into two main tasks: diffusion-type classification at the whole-trajectory level and temporal segmentation of dynamic states within individual trajectories.

### 4.1 Diffusion-Type Classification

Over the years, a variety of stochastic models have been developed to describe deviations from classical Brownian motion in SPT data. Some of the most commonly studied diffusion models in SPT include: Brownian motion (normal diffusion), fractional Brownian motion (FBM),^84^ continuous-time random walk (CTRW),^85,86^ and Lévy walks (LW).^87^ Accurately differentiating these diffusion types offers important insights into the underlying biophysical mechanisms. Traditionally, such classification has depended on handcrafted statistical descriptors, including the time-averaged mean squared displacement (TAMSD), velocity autocorrelation function (VACF),^88^ power spectral density (PSD),^89,90^ and p-variation analysis.^91^ These methods are physically interpretable and perform well under idealized conditions. However, they often rely on assumptions of stationarity and trajectory homogeneity, which make them vulnerable to noise, short track lengths, and dynamic heterogeneity. These factors are often unavoidable in real experimental data.

Machine learning has gained increasing attention as a data-driven framework for diffusion analysis in single-particle tracking. Unlike classical methods that rely on predefined physical models or handcrafted summary statistics, machine learning approaches can automatically learn informative features from raw trajectories, making them well-suited to capture the diversity and complexity of intracellular dynamics.^92^ Early applications of machine learning in this domain typically followed a two-step paradigm: first, manual extraction of descriptive features from particle trajectories and, second, feeding these features into conventional classifiers such as random forests. This approach combines domain knowledge to design interpretable features with the generalization capabilities of statistical learning algorithms. A representative and influential example of this paradigm is the diffusional fingerprinting framework proposed by Pinholt et al.,^54^ which extracted a comprehensive set of 68 statistical features (including mean square displacement, asymmetry, ergodicity breaking parameters, and displacement distributions) and used them as inputs to the logistic regression model (Figure 7). This work demonstrates that a sufficiently rich set of handcrafted statistical descriptors can enable accurate and interpretable classification of heterogeneous diffusion behaviors without the need for explicit physical modeling. Furthermore, by introducing the concept of a “diffusional fingerprint,” the authors established a unified feature representation that supports cross-system comparisons and offers insights into the physical mechanisms governing particle motion.

**Figure 7:**
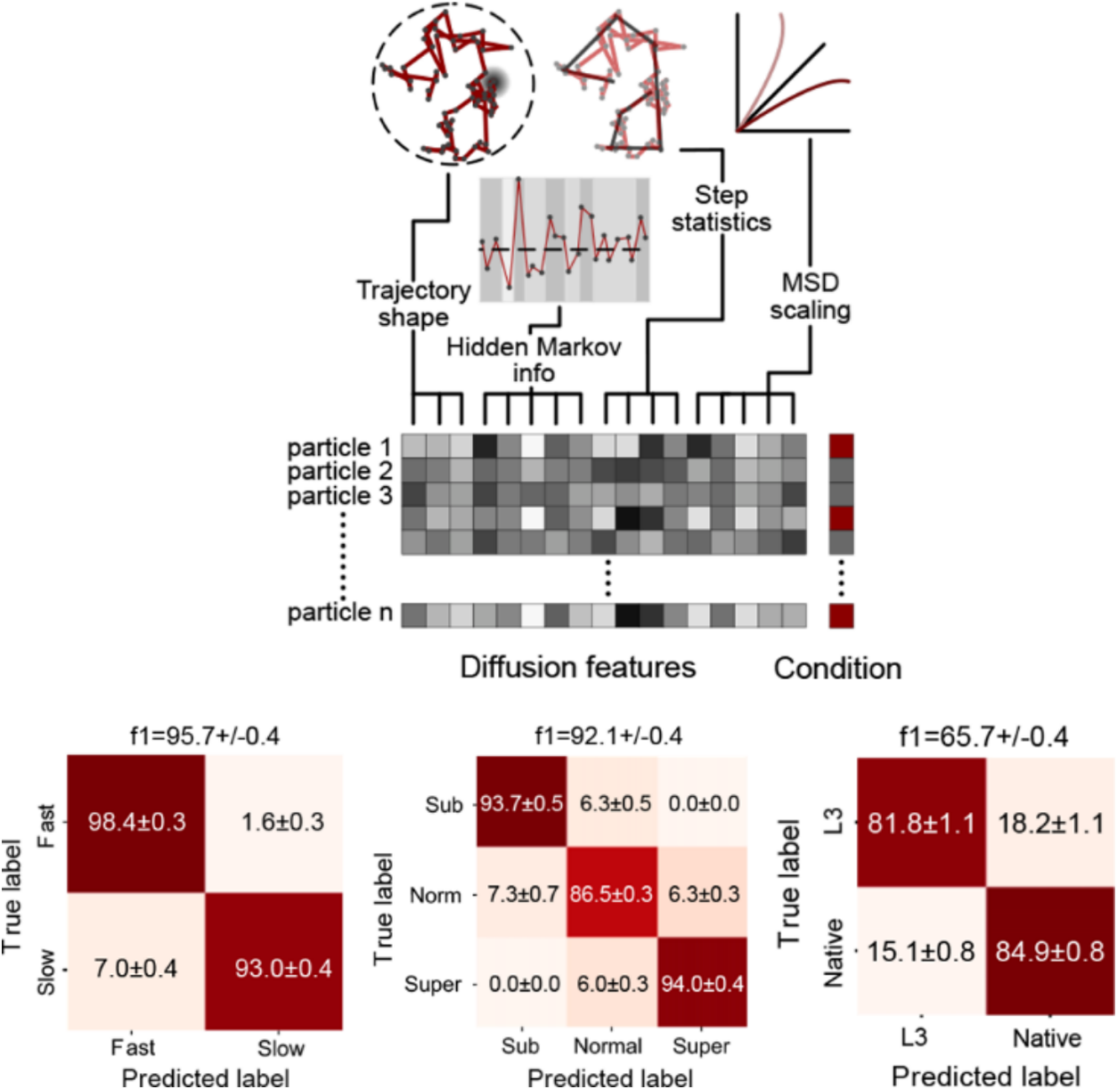
Diffusional fingerprinting for SPT trajectory analysis introduced by Pinholt et al.^54^ Top: Workflow for constructing a “diffusional fingerprint”: for each trajectory, a compact set of descriptors is computed: HMM-based residence times/occupancies (state-shifting behavior), MSD scaling (α, D), step-statistics, trajectory-shape metrics (e.g., trappedness, fractal dimension, efficiency), and Gaussianity—then aggregated across conditions to form a feature matrix. Bottom: Representative classification results using a simple logistic regression classifier: (left) discrimination of speed-switching trajectories (fast vs slow); (middle) separation of subdiffusive, normal, and superdiffusive motion; and (right) distinction between two enzyme variants (L3 vs native lipase) from experimental data. Heatmaps show per-class performance (confusion matrices). Copyright 2021 Proc. Natl. Acad.

In recent years, deep learning has become a powerful tool for diffusion state identification, offering strong nonlinear modeling capabilities, automatic feature extraction from high-dimensional and complex temporal data, and robustness in detecting heterogeneous diffusion patterns. Unlike conventional approaches that depend on predefined dynamic models or handcrafted features, deep learning provides an end-to-end framework for inferring latent dynamic structures directly from raw trajectory coordinates. This is particularly advantageous for modeling non-Gaussian, nonstationary, and multistate hybrid diffusion processes.

Among deep learning architectures applied to diffusion-type classification, convolutional neural networks (CNNs)^93,94^ and recurrent neural networks (RNNs)^95,96^ are the most widely adopted and extensively validated. CNN approaches operate either on 1D coordinate sequences^50^ (Figure 8a) or on full 2D trajectories49 (Figure 8b) to classify diffusion modes and estimate key parameters (e.g., the FBM Hurst exponent and Brownian diffusion coefficients). Across simulated benchmarks, these end-to-end models typically surpass feature-engineered baselines in accuracy and noise robustness, especially for short or low-SNR trajectories. In parallel, RNNs capture temporal dependencies: multitask LSTM frameworks jointly classify, regress parameters, and segment trajectories^97^ (Figure 8c), while comparative studies find LSTM/GRU variants outperform simple RNNs and statistical baselines across track lengths and noise levels^98^ (Figure 8d). Collectively, deep networks mitigate nonstationarity and heterogeneity and are establishing new performance standards for diffusion-type classification.

**Figure 8:**
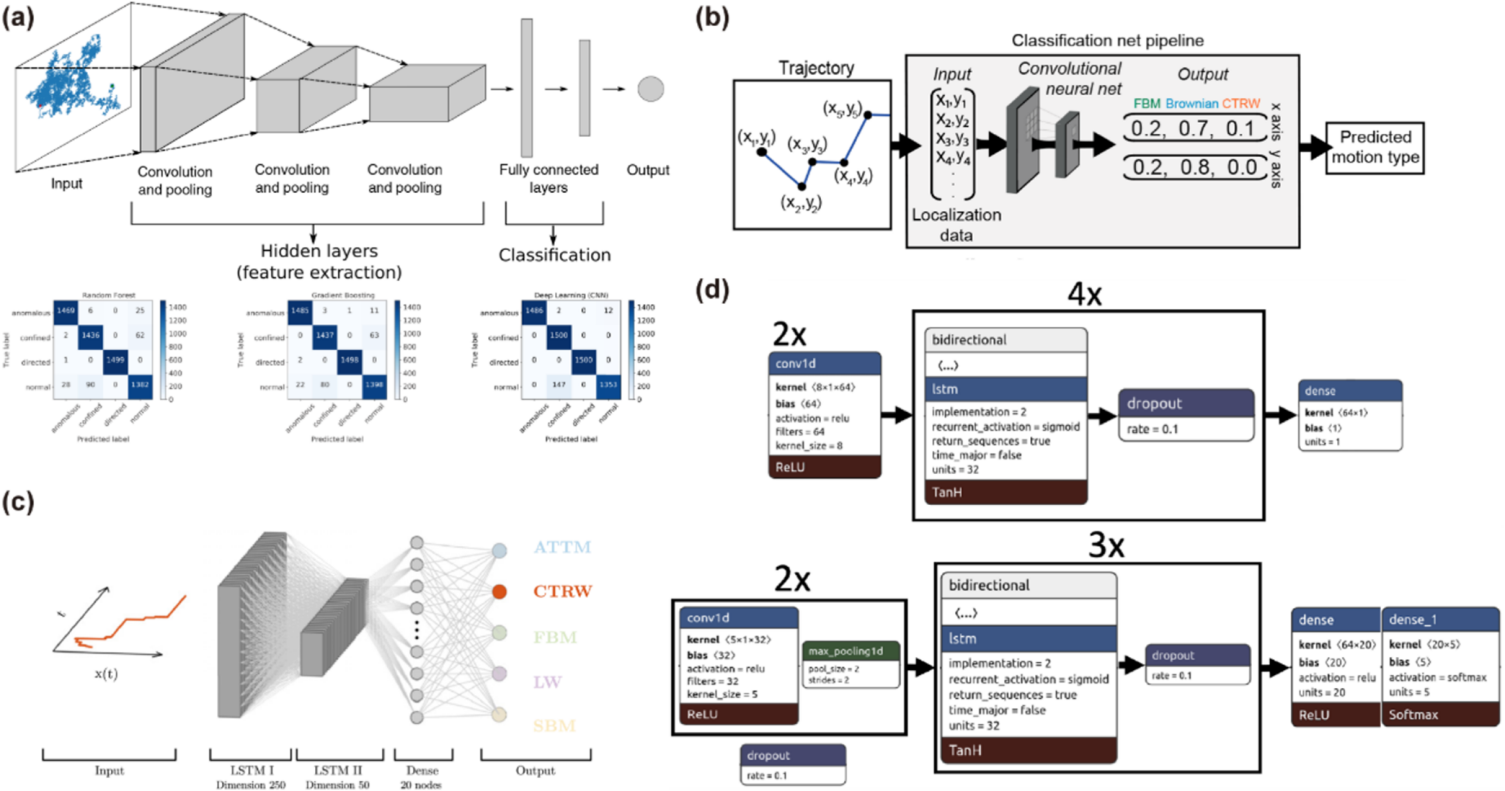
Applications of deep learning in diffusion-type classification. (a) Schematic architecture of a CNN (top) and a comparison of the performance of two machine learning methods with CNN (bottom). Adapted with permission from ref 50. Copyright 2019 American Physical Society. (b) Schematic representation of the classification using CNN. Adapted with permission from ref 49. Copyright 2019 Elsevier. (c) Structure of the RANDI model is used for identifying the anomalous diffusion trajectories, which has two layers of LSTM. Adapted with permission from ref 97. Copyright 2021 IOP Publishing. (d) Schematic of two different model architectures using LSTM, in which one is used for regression (top), and the other one is used for classification (bottom). Adapted with permission from ref 98. Copyright 2007 IOP Publishing.

### 4.2 Diffusion State Identification

While classifying entire trajectories into diffusion types provides an initial understanding of molecular motion, diffusion behavior in real biological systems often exhibits pronounced spatiotemporal heterogeneity. At different time points, particles may be influenced by local microenvironmental factors such as the cell membrane, cytoskeleton, or protein complexes, leading to transitions between distinct diffusion states. Accurately identifying these latent diffusion states is essential for elucidating molecular dynamics and the underlying biochemical processes within cells.

Early trajectory analyses often employ sliding-window techniques to detect local changes in the diffusion behavior. In these approaches, local diffusion parameters are estimated along the trajectory to identify segments with distinct mobility. This window-based segmentation offered an initial view of state heterogeneity by revealing regions of confined diffusion within a single trajectory. However, the accuracy of sliding-window method is constrained by their sensitivity to noise and by the choice of window size, which can obscure rapid state transitions or fragment longer steady-state periods.^43,57^

HMM provides a rigorous framework for identifying diffusion states, treating them as latent variables defined by parameters, such as diffusion coefficients or motion modes. Fitting an HMM to trajectory data simultaneously segments trajectories and estimates state parameters and transition probabilities. However, conventional HMMs require the number of states to be specified in advance and often assume memoryless Brownian motion, which can cause underfitting, overfitting, or failure to capture long-term correlations and anomalous transport. To address these limitations, researchers have developed more flexible Bayesian extensions. For example, the variational Bayesian HMM (VB-HMM) replaces point estimates with posterior distributions over model parameters through variational inference, increasing robustness to noise and reducing overfitting.^99^ The infinite HMM (iHMM) uses a nonparametric Bayesian framework to automatically infer the number of hidden states from the data, removing the need for manual specification and improving the adaptability to complex trajectories. In addition, hybrid models that combine Gaussian mixture models (GMMs) with HMMs have been introduced to enhance initialization or improve the modeling of observation distributions.^47^ In these methods, GMMs first estimate the number or type of diffusive states based on displacement statistics, and HMMs then infer the most probable sequence of state transitions. This combination helps to reconcile rigid model assumptions with empirical data, particularly when state boundaries are difficult to define.

Bayesian extensions offer greater model flexibility but still depend on probabilistic assumptions and can be computationally demanding. Several studies use a two-step approach to identify diffusion states: trajectory data are segmented into short fragments, statistical features are extracted, and these are classified with methods such as random forests or support vector machines. This strategy balances interpretability with the power of established classifiers and has been effective in tasks like detecting viral particle motion patterns^100^ or incorporating features from hidden Markov models.^101^

The 2021 Anomalous Diffusion (ANDI) Challenge showcased state-of-the-art algorithms for tasks, including diffusion model classification, parameter regression, and trajectory forecasting. Building on their success in classifying diffusion types, CNNs and RNNs have been adapted to capture the greater temporal complexity and resolution needed to identify dynamic state transitions within single trajectories.

Among RNN-based approaches, DL-MSS was one of the first deep learning models for anomalous diffusion analysis, using LSTM networks to classify diffusion behaviors^102^ (Figure 9a). It introduces the moment scaling spectrum (MSS), a local descriptor that captures higher-order statistical features of particle trajectories and feeds them into a two-layer LSTM for sequence classification. This design improves the interpretation of local dynamics and reveals how drug treatments affect molecular motion. However, DL-MSS depends on fixed-size, manually segmented trajectory windows and assumes homogeneity within each segment, limiting its ability to detect gradual or continuous transitions. Building on this, NOBIAS combines nonparametric Bayesian modeling with LSTM-based classification^103^ (Figure 9b). This hybrid framework overcomes two major limitations of earlier HMM- and segment-based methods: the need to predefine the number of hidden states and the inability to capture complex, nonlinear spatiotemporal dynamics. By inferring the number and parameters of latent states and classifying each segment into canonical diffusion types, NOBIAS improves segmentation accuracy under heterogeneous conditions.

**Figure 9:**
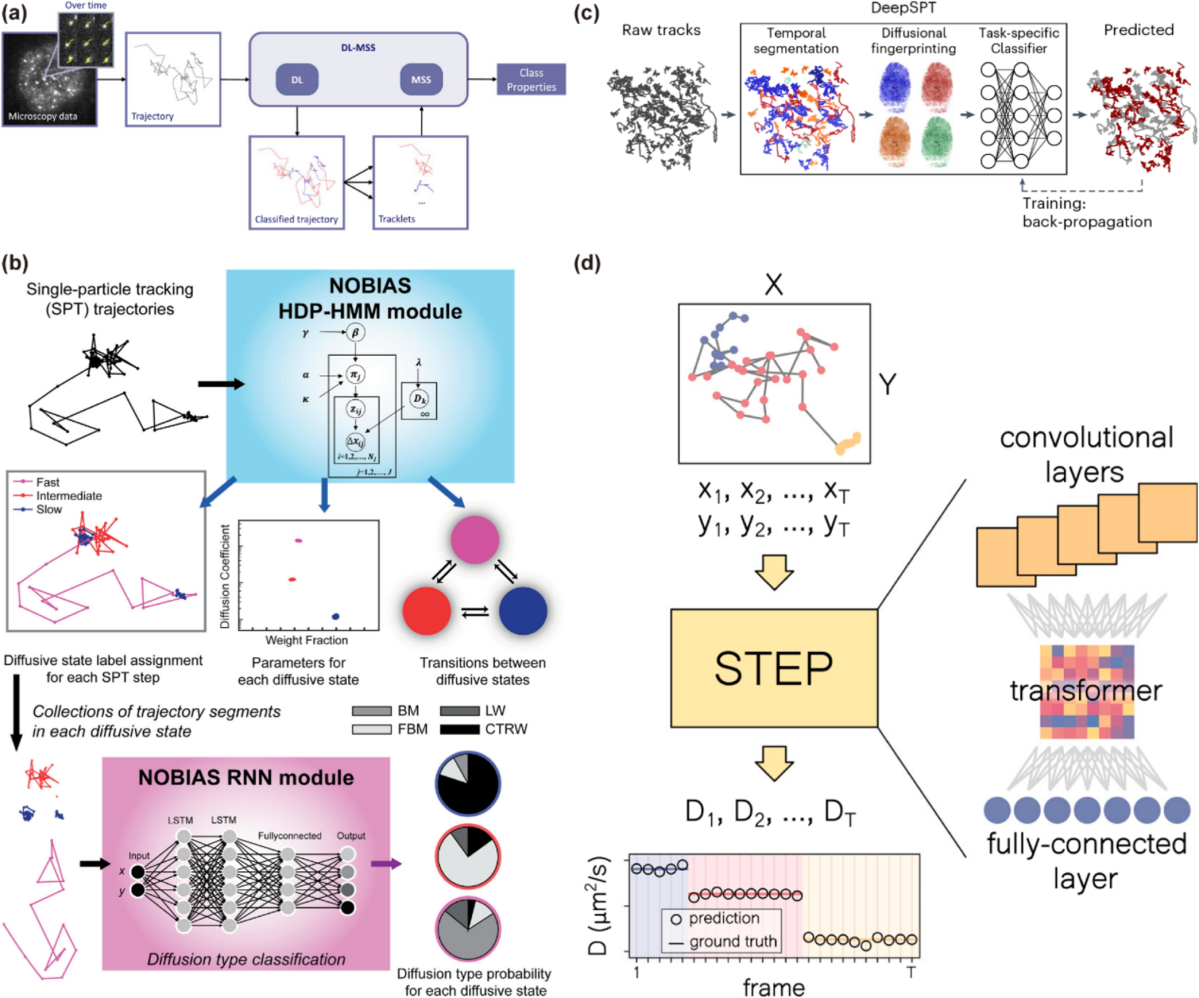
Supervised deep learning methods for diffusion state identification. (a) Schematic diagram of the DL-MSS method. Adapted with permission from ref 102. Copyright 2019 Springer Nature. (b) Workflow of NOBIAS, in which two modules process the trajectories in sequence. Adapted with permission from ref 103. Copyright 2021 Frontiers. (c) Schematic representation of the DeepSPT classification pipeline. Adapted with permission from ref 42. Copyright 2025 Springer Nature. (d) Illustration of the STEP pipeline. Adapted with permission from ref 104. Copyright 2023 Cell Press.

In the CNN-based line, DeepSPT integrates U-Net with a 40-feature “physical fingerprint” module and multitask predictors,^42^ linking motion dynamics to biological function and pioneering a “motion-to-function” paradigm (Figure 9c). It generalizes well across biological contexts but relies heavily on labeled data and engineered features, which may limit the adaptability to new systems. To address the constraints of discrete segmentation, Requena et al. developed the STEP model,^104^ a CNN-transformer sequence-to-sequence model that performs frame-level regression of physical parameters (Figure 9d). STEP models heterogeneous diffusion as a continuous process, capturing gradual transitions without imposing state boundaries. This represents a significant departure from traditional segmentation-based approaches.

While supervised deep learning methods perform well in diffusion state identification, they are constrained by the need for large labeled data sets, often generated through simulations. Unsupervised learning offers a powerful alternative, particularly when labels are scarce or ambiguous. Among recent advances in unsupervised diffusion state identification, Deep-SEES represents a significant step toward leveraging deep learning for data-driven representation learning^105^ (Figure 10). Instead of relying on handcrafted features or predefined motion categories, it employs a GRU-based sequence encoder to extract latent embeddings of particle dynamics, followed by an experience-guided segmentation module that refines state boundaries via iterative clustering. This approach captures both abrupt and gradual transitions, making it well-suited to heterogeneous intracellular environments, although it still requires parameter tuning. Kabbech et al. introduced another framework inspired by the Noise2Noise paradigm,^106^ which predicts expected displacement magnitudes without labels or simulation-based pretraining. It matches the performance of leading supervised methods, even on experimental data, and models dynamics continuously without segmentation.

**Figure 10:**
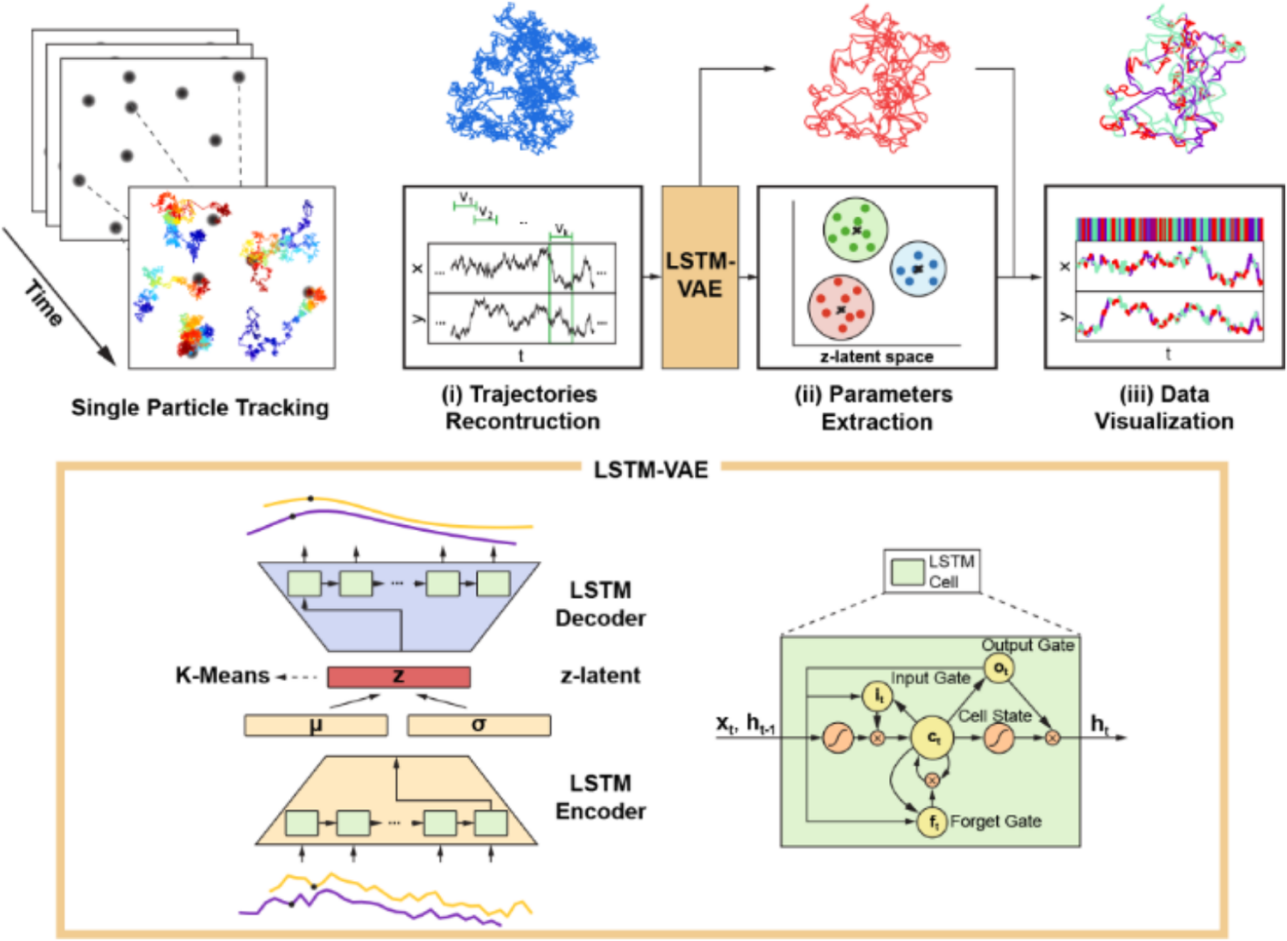
Deep-SEES for unsupervised diffusion state discovery from SPT trajectories. Top: End-to-end workflow: raw single-particle tracks are reconstructed into time-series; overlapping subtrajectories are embedded by an LSTM-VAE to learn a noise-robust latent representation; latent vectors are clustered to extract dynamical states and their transitions; per-state parameters and frame-level labels are then visualized along each trajectory. Bottom: Network design of the LSTM-VAE used in Deep-SEES: a two-layer LSTM encoder maps subtrajectories to a Gaussian latent space; samples are decoded by a symmetric LSTM to reconstruct denoised subsequences; a k-means-regularized objective shapes the latent space for separable state clusters. Adapted with permission from ref 105. Copyright 2023 Elsevier.

In summary, while traditional models offer valuable physical interpretability, they often struggle with the heterogeneity and noise of the experimental trajectories. Machine learning improves predictive accuracy and fundamentally changes how diffusive behavior is defined and detected. The key challenge ahead is balancing predictive performance with interpretability to ensure that these methods remain biologically relevant. Representative machine learning methods for diffusion-type classification and diffusion state identification are summarized in Table 1.

**Table 1:** Summary of Machine Learning Methods

method	task^a^	model^b^	input^c^	supervised/unsupervised^d^	training data source^e^	handcrafted features^f^	level of interpretability^g^
Kowalek et al.^107^	1	XGBoost	Features	Supervised	Simulated	Yes	Moderate
Janczura et al.^51^	1	Random Forest + Gradient Boosting	Features	Supervised	Both	Yes	Moderate
Pinholt et al.^54^	1	Logistic Regression	Features	Supervised	Both	Yes	High
Kowalek et al.^50^	1	CNN	1D Trajectory	Supervised	Simulated	No	Low
Granik et al.^49^	1	CNN	2D Trajectory	Supervised	Both	No	Low
RANDI^97^	1/2	LSTM	1D/2D/3D Trajectory	Supervised	Simulated	No	Low
Garibo et al.^98^	1	CNN + LSTM	1D/2D/3D Trajectory	Supervised	Simulated	No	Low
Matsuda et al.^47^	2	GMM + HMM	Squared Displacement	Unsupervised	\	No	High
Helmuth et al.^100^	2	SVM	Features	Supervised	Both	Yes	Moderate
Malkusch et al.^101^	2	Random Forest + SVM	Features	Supervised	Both	Yes	Moderate
DL-MSS^102^	2	LSTM	2D Trajectory	Supervised	Both	No	Low
NOBIAS^103^	2	HD-HMM + LSTM	2D Trajectory	Supervised	Both	No	Moderate
DeepSPT^42^	2	U-net + CNN	2D/3D Trajectory	Supervised	Both	No	Low
STEP^104^	2	CNN + Transformer	2D/3D Trajectory	Supervised	Both	No	Low
Deep-SEES^105^	2	LSTM-VAE	2D Trajectory	Unsupervised	\	No	Low
Noise2Noise^106^	2	LSTM	2D Trajectory	Unsupervised	\	No	Low

a Methods are categorized according to their application—either diffusion-type classification (1) or diffusion state identification (2).

b The machine learning framework or algorithm used in the study.

c The type of data or features used as input to the model.

d Specifies whether the method relies on labeled data for training.

e Indicates whether the model was trained using simulated data, experimental data, or both.

f Whether manual feature extraction or engineering was required before training (Yes/No);

g The extent to which the model’s decision process can be explained, categorized as high, moderate, or low.

## 5 Uncertainty and Bias of ML Models in the SPT

One incentive to apply ML/DL methods in SPT is that these approaches enable the analysis of large data sets while mitigating human biases commonly encountered in the interpretation of SPT data. However, it is also important to recognize that these models can introduce model-associated biases from insufficient training data, overfitting or underfitting, and inherent algorithmic assumptions that may not fully capture the complexity of particle dynamics. This is especially important for SPT as these experiments often yield short, noisy, and heterogeneous trajectories that are of mixed states and are difficult to characterize using a limited number of parameters, making it challenging to interpret diffusion behavior with confidence. Quantification of uncertainty is, therefore, crucial when applying ML models to SPT data.

In conventional SPT, estimating uncertainty is relatively simple as position estimation or analysis was often deterministic analytical method. For example, the Cramér-Rao lower bound provides a theoretical minimum variance dependent on photon count, background noise, and PSF scale for unbiased estimators, offering a baseline for localization precision.^108^ The uncertainty of MSD originates from the finite length of trajectories and measurement noise. To address this, it is common to compute ensemble averages of multiple trajectories^109^ or to compute MSD versus time lag representing only a portion of a given trajectory.^110^

Simpler ML models such as feature-based classifiers or regressors naturally provide measures of confidence. For example, Pinholt and co-workers proposed a “diffusional fingerprinting” framework, which uses 17 statistical features per trajectory and a logistic regression to classify diffusion behavior.^54^ This approach achieves high accuracy across diverse experiments and inherently produces a probability for each predicted class, indicating the model’s certainty. Janczura and colleagues^51^ systematically compared Random Forest (RF, Figure 11a) and Gradient Boosting (GB) ensembles using a novel feature set to classify SPT trajectories into Brownian, subdiffusive, and superdiffusive regimes, demonstrating 94% accuracy on both synthetic benchmarks and real GPCR data. They showed that these conventional ML models naturally yield predictive class probabilities, providing direct measures of uncertainty without additional postprocessing.

**Figure 11:**
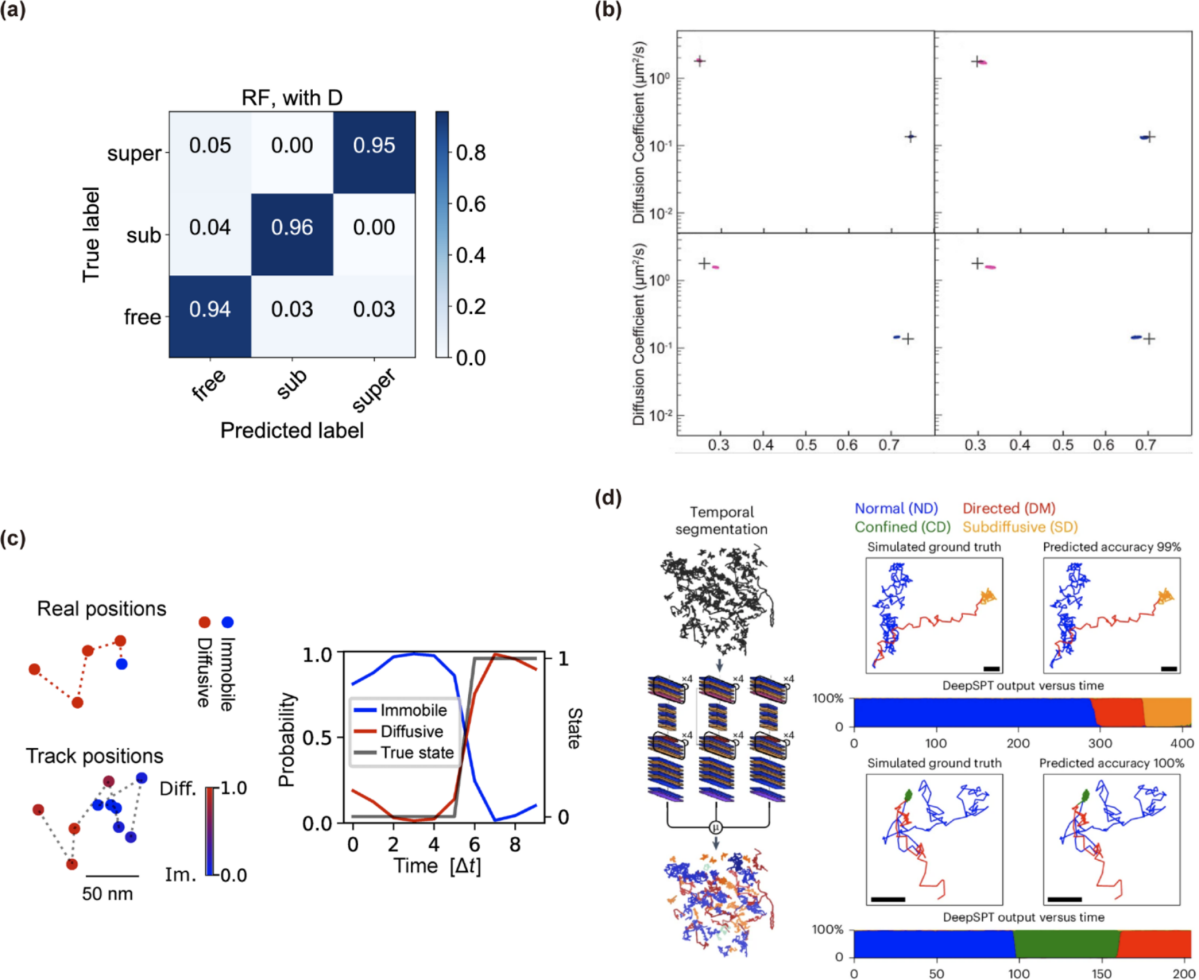
Uncertainty quantification in ML/DL SPT methods. (a) Row-normalized confusion matrix for a Random Forest classifier reported by Janczura et al.; a matched model without diffusion coefficient D shows nearly identical diagonals, indicating robustness and that predictions are not dominated by a single feature. Adapted with permission from ref 51. Copyright 2020 American Physical Society. (b) Posterior uncertainty from NOBIAS on a two-state mixture. Dots show posterior samples of each state’s mean apparent diffusion coefficient vs weight fraction; black crosses mark ground truth. Upper-left: no blur, abundant data (500 × 100-step); upper-right: no blur, sparse (2,000 × 10-step); lower-left: motion blur, abundant; lower-right: motion blur, sparse. Adapted with permission from ref 103. Copyright 2021 Frontiers. (c) Example SPT trajectory with transitions between states inferred by ExTrack: circles show true positions and arrows the observed displacements; frames colored blue (immobile/bound) or red (diffusive) indicate the MAP state path derived from per-time point state probabilities, providing an uncertainty-aware segmentation used downstream for dwell-time and rate estimation. Adapted with permission from ref 120. Copyright 2023 Rockefeller University Press. (d) Per-frame state-probability traces from DeepSPT’s segmentation module (normal, directed, confined, subdiffusive), calibrated by temperature scaling. Adapted with permission from ref 42. Copyright 2025 Springer Nature.

For diffusion parameter estimation, fully Bayesian treatments have been demonstrated to yield rigorous measures of uncertainty. For example, El Beheiry et al. introduced InferenceMAP,^111^ a Bayesian framework that maps spatially varying diffusion coefficients and outputs full posterior distributions for each local estimate. Similarly, Thapa et al. applied nested-sampling Bayesian inference to synthetic and real SPT data sets, explicitly computing posterior distributions over diffusion parameters and quantifying their credible intervals.^112^ HMMs are also a powerful tool in uncertainty quantification, as they can provide a confidence measurement for every state assignment. Monnier et al.^44^ extended HMMs to include directed transport and used Bayesian model selection to annotate trajectories with single-step resolution, yielding per-step probabilities over diffusive vs transport states.

In DL applications, however, estimating uncertainty is not always as intuitive. Several factors make it difficult to quantify uncertainty in DL models, and there are unique challenges specific to SPT data. These include the lack of uncertainty output, insufficient training data, difficulty in detecting out-of-domain data, and the stochastic and complex nature of SPT data.

Conventional DL models often struggle to give reliable uncertainty outputs because they lack probabilistic foundations.^113^ Deep neural networks are typically trained as point estimate function approximators (via minimization of a loss) rather than as full probabilistic models, so they do not natively provide uncertainty estimates. Liu et al.^114^ formalize this gap and demonstrate that a standard DNN, without any Bayesian machinery or ensembles, cannot quantify its predictive uncertainty. It is common to treat the output of a softmax layer as a “confidence” score, but these scores do not reliably correspond to the true probability of correctness;^115^ contemporary deep classifiers tend to be overconfident in their decisions.^116^ Typical networks produce a point estimate with no error bar; therefore, there is no built-in mechanism for predictive intervals. The lack of a formal weight posterior means epistemic uncertainty (model uncertainty) is ignored unless special Bayesian methods are used.^10^

One powerful approach to modeling uncertainty in SPT is the use of nonparametric Bayesian inference, exemplified by the hierarchical dirichlet process hidden Markov model (HDP-HMM). One of the key innovations of NOBIAS is its data-driven identification of both how many and what kinds of diffusive states are present rather than relying on any a priori labels (Figure 11b). Although the field has increasingly recognized that fixing the state count can introduce serious bias, it is even more common, and equally restrictive, to predefine state types (e.g., subdiffusive, normal, and superdiffusive). That approach can easily obscure meaningful distinctions between different behaviors of subtle differences, as in this current case, where Chen and colleagues applied NOBIAS to tracks from *Bacteroides thetaiotaomicron* and resolved two separate slow-diffusion regimes, in which one with anomalous exponent α_1_ = 0.38 and another with α_2_ = 0.46 on top of a fast-diffusing state. The two slow states with symmetric diffusion coefficients along both *x* and *y* axis indicate the existence of two states with different degrees of engagement and were aligned with previous study of starch starvation.^117^ In comparison, similar diffusion state quantification methods like SMAUG^118^ and vbSPT,^99^ yielded 4 and 10 diffusion states, respectively, resulting in significant hardship in drawing biologically relevant interpretations. It is noticeable that vbSPT, although accounting for uncertainty via variational Bayesian inference, underestimates model uncertainty and parameter variance. Its mean-field approximation locks in a suboptimal K by overconfident evidence maximization, failing to reflect the probability of adjacent state counts. Additionally, reliance on conjugate-prior updates biases diffusion parameters when trajectories are short, yielding narrow credible intervals. Consequently, vbSPT misinterprets sampling noise as distinct states, fragmenting trajectories into too many diffusive modes. Sun and Paninski^119^ demonstrated that an amortized Bayesian neural network can perform multiparticle tracking while outputting the probabilistic uncertainty in each particle’s location and even in the identity assignment when tracks come close. Such a method retains the uncertainty information that traditional deterministic trackers would discard, delivering probabilistic “error bars” on particle positions and trajectories. As a similar approach, Simon et al. proposed ExTrack^120^ (Figure 11c), which treats state assignment probabilistically: after fitting global parameters, it computes per-time point posteriors and annotates each frame with these probabilities. For downstream kinetics, ExTrack constructs state-duration distributions from the probabilistic annotations to flag hidden or non-Markovian behavior and guide model choice, and it refines positions with associated variance—reducing localization error by ∼1/√N. Complementary to probabilistic HMMs like ExTrack, DeepSPT is a task-oriented DL pipeline that targets rapid, automated biological readouts from SPT (Figure 11d). In virus-entry case studies, it mapped functional states in seconds rather than weeks, reporting F1 scores of 81%, 82%, and 95% for endosomal organelles, clathrin-coated pits, and vesicles, respectively; however, the framework emphasizes throughput and accuracy rather than formal Bayesian uncertainty, so class scores should be calibrated and stress-tested for OOD data.

Another approach to achieve reduced bias and uncertainty-aware SPT is via self-supervised latent-space segmentation. Deep-SEES^105^ is another example that advances uncertainty quantification in SPT by embedding each trajectory segment into a probabilistic latent space via a variational LSTM autoencoder, rather than mapping it to a single point estimate. During training, the encoder learns both mean and variance for each subtrajectory’s latent vector, such that broader variances directly signal ambiguous or mixed-state behaviors, while tighter distributions indicate well-resolved dynamics. Like NOBIAS, Deep-SEES autonomously discovers the number and nature of dynamic states by clustering these learned embeddings, revealing confined, Brownian, and directed transport modes solely from the data and requires no a priori labels or predefined diffusion models. When applied to trajectories from PEGylated AuNRs interacting with catalytic enzymes, it uncovered 3 states, including a superdiffusion state, a subtle enzyme-mediated transport phenomena that previous methods^121^ overlooked. While we develop methods to mitigate bias, it is important to realize the source of bias and handle or remove it before designing the workflow. In many cases, DL models were trained exclusively on simulated data, whether self-generated^51,52,122^ or existing simulated data sets, e.g., the AnDi challenge.^123–125^ Gajowczyk and colleagues noted that a convolutional neural network trained on simulated data struggled to generalize to trajectories from different sources.^126^ In some cases, although experimental data were used to train models, the scenarios are restricted to highly specific contexts, making them vulnerable to poor predictions and high uncertainty. For example, Song et al.^59^ developed a DNN model to infer the position and angle of anisotropic gold nanorods from biplane images collected in living cells under a particular imaging setup. While highly effective within its intended domain, this model would likely exhibit high uncertainty and produce poor predictions when applied to out-of-domain (OOD) scenarios such as different probes, cell types, or imaging setups. This can be especially challenging to users without significant exposure to ML models, as the models do not report uncertainty or confidence.

One common factor that introduces bias is that real data are heterogeneous and the source of uncertainty is complex. For example, in diffusion state classification, one of the most frequently visited problems in SPT, the uncertainty in attribution of states can be both aleatoric and epistemic. A diffusing particle at a given time bears a mixture of diffusion states.^127^ In most cases, models tend to attribute a particle to a deterministic state without reporting confidence or likelihood.^128^ Such treatment lacks a consideration of complexity in living systems and introduces aleatoric uncertainty. A particle can also undergo different diffusion states in a trajectory, while some models lack the ability to segment trajectories into distinct intervals for analysis.^54^ This is an example of epistemic uncertainty. It is noticeable that even if significant attention is given to mixture or transition between diffusion states, ML models can fail to adequately describe biological systems, as these models ultimately model trajectories into well-defined diffusion states, and these states may not be representative in real-world problems.

## 6 Inferring Molecular States and Stoichiometry with ML

Many important biological processes rely on molecular transitions between dynamic states, and SPT techniques have played an essential role in characterizing the state transitions involved in these processes such as binding and unbinding between ligands and receptors,^129–131^ assembling into complexes,^120,132,133^ being actively transported,^134,135^ or undergoing conformational changes.^136,137^ These state transitions alter a molecule’s mobility and interactions, which in turn regulate function. A key strength of SPT is its ability to resolve heterogeneous and transient behaviors that are averaged out in ensemble measurements. For example, Oviedo-Bocanegra and colleagues^138^ observed that the diffusion of membrane-anchored endoribonuclease RNase Y consisted of a roughly even mixture of slow/static population and faster population in exponentially growing cells, while the previous ensemble epifluorescence study^139^ could only resolve a single state.

Traditional SPT analyses rely on manually classifying trajectory patterns or fitting simple statistical models to trajectory data. These approaches have proven useful for inferring motion modes^140^ and describing diffusion-associated physiology.^141^ However, as systems become more complex, the limitations of traditional SPT analysis become apparent. Manual interpretation of the trajectories is inherently subjective and prone to human bias. The MSD curve is often used to determine the type of diffusion. Plateaus of MSD curves are commonly interpreted as indicators of confinement and used to quantify the size of such confinement. For example, Wu and colleagues observed that store depletion led to a dramatic reduction in the plateau of time-averaged MSD of STIM1-GFP, consistent with a ∼4–5× decrease in the radius of gyration and indicative of much tighter confinement of STIM1-Orai1 complexes at ER-PM junctions.^142^ While useful, the MSD has significant limitations. Typically, lag time cannot exceed 20% of total trajectory duration,^143^ which requires very long trajectories to observe plateaus. MSD is also unreliable when it comes to dynamics at small time scales^144^ and has poor performance at low-SNR conditions.^145^ Methods that involve manual interpretation of data, which is common for MSD-based analysis, are not scalable as microscopic systems can easily generate thousands of data sets.^146^

ML and DL methods, on the other hand, can fill the gap of conventional statistical methods. A straightforward way to improve upon these traditional analyses is to use simple, supervised ML classifiers to assign an overall diffusion type to each trajectory based on a small set of physically meaningful features (e.g., anomalous exponent, apparent diffusion coefficient, standardized maximum displacement, and statistical test *p*-values). These models require minimal feature engineering and can be trained on synthetic data where the ground truth motion type is known and then directly applied to experimental data sets. For example, Janczura and colleagues trained RF and GB models to classify trajectories as subdiffusive, normal diffusive, or superdiffusive, and validated them on live-cell GPCR tracking data.^51^ The models correctly reproduced the expected predominance of sub- and normal diffusion in crowded membranes, with almost no superdiffusion detected. Granik et al. reported a simple DL model that trains on 300k simulated trajectories with known ground truth labels to classify entire trajectories into Brownian, FBM, or CTRW modes.^49^ On experimental data obtained from 100 and 200 nm polystyrene beads diffusing in a 40% glycerol–water solution, they achieved the same confidence interval as ensemble-MSD with ∼1/2 the number of trajectories, and predicted diffusion coefficients were aligned with values predicted by the Stokes–Einstein equation. Even though such classifiers treat each trajectory as homogeneous and do not account for intratrajectory state changes, they already provide significant benefits by removing subjective bias, standardizing classification criteria, and operating robustly on large data sets with minimal user intervention. As a result, they represent a practical first step toward more sophisticated state-resolved analyses.

A major challenge in understanding the biology encoded in SPT data is that single trajectories can contain transitions between diffusive states, requiring methods capable of identifying an arbitrary number of heterogeneous states over time. An important and often simpler subset of the problem involves 1D time-series data such as photobleaching intensity traces or single-coordinate position estimates treated independently. These data sets share the single-molecule, time-resolved nature of full SPT trajectories but are typically easier to probe and interpret, as the analysis often focuses on detecting discrete plateaus and transitions rather than modeling complex multifeature diffusion behaviors. In single-molecule stoichiometry analysis, early algorithms such as AutoStepfinder^147^ (Figure 12a) illustrate what can be achieved without machine learning. The algorithm iteratively partitions 1D intensity traces and uses an S-curve criterion to select the optimal number of steps; it is fast and unbiased but tends to oversegment noisy traces. Benchmarking by Bandyopadhyay and colleagues^148^ against other unsupervised idealization methods shows that AutoStepfinder consistently yields lower F1 scores because it overfits noise fluctuations and misidentifies short dwells. To overcome these limitations and handle heterogeneous photobleaching events, supervised ML approaches have been proposed. Xu and co-workers^45^ trained a convolutional/LSTM deep neural network (CLDNN) on ∼50,000 synthetic traces plus ∼15,000 labeled experimental traces to count 0–4 bleaching steps, achieving >90% accuracy at SNR ratios around 2 and completing analyses roughly 2 orders of magnitude faster than manual or HMM-based methods. Wills et al.^149^ introduced FluoroTensor, which directly builds on CLDNN but removes a key practical constraint: FluoroTensor’s compact convolutional recurrent neural network is not limited by a minimum plateau length and can detect bleaching events even in consecutive frames, including the first frame of observation. Its step-counter uses 96% fewer parameters than the prior state-of-the-art model while improving accuracy, and its CNN step-localizer fits >90% of step times within one frame of ground truth. Performance of FluoroTensor scales with SNR (from 74% accuracy at SNR≈1.105 to 98.1% at SNR≈8.853). In head-to-head simulations, FluoroTensor outperformed CLDNN and a maximum-likelihood (MLE) baseline overall.

**Figure 12:**
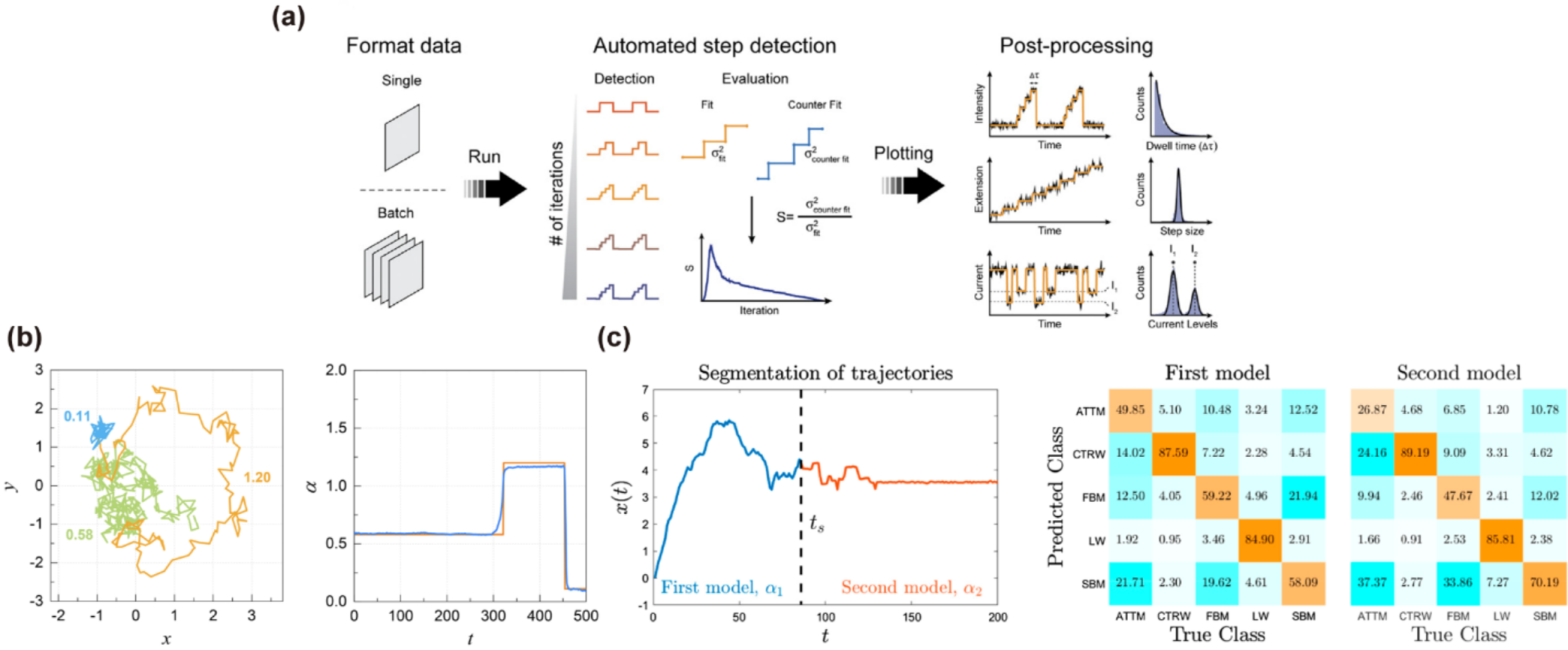
ML/DL applications in segmentation and state identification in SPT (a) workflow of AutoStepfinder to classify discrete stoichiometric transitions. Adapted with permission from ref 147. Copyright 2021 Cell Press. (b) Left: a 2D trajectory with color-coded segments; numbers indicate the anomalous exponent α estimated for each segment predicted by U-AnDi. Right: α­(t) for the same track. Adapted with permission from ref 155. Copyright 2024 American Physical Society. (c) RANDI recurrent-network segmentation and model identification. Left: time-series of the anomalous exponent α­(t) for a trajectory switching between two diffusion models. Right: segment-wise confusion matrices for diffusion-model ID. Adapted with permission from ref 97. Copyright 2021 IOP Publishing.

Compared to 1D intensity traces, deciphering molecular states from SPT trajectory data is uniquely challenging, as results from plateau-identification are not informative in 2D or 3D trajectories. Previously, we have shown that manual segmentation and attribution of diffusion states is labor-intensive, prone to user bias and inconsistencies, and infeasible for large data sets, and automated trajectory-wise classification (assigning a single label to each whole trajectory), whether rule-based or simple machine learning, ignores the fact that a particle can undergo mixed diffusion states inside a track. Therefore, reliable segmentation is often necessary for trajectories obtained from context-sensitive experiments. When performing trajectory segmentation, it is important to evaluate how the number of distinct states is obtained and reported.

There are two ways to describe intertrajectory heterogeneity: by reporting whole-trajectory state-associated parameters (e.g., diffusion coefficient or abnormality factor) or by modeling discrete state switching and explicitly segmenting a trajectory into different modes and reporting parameters in each interval. Assuming multiple states while not explicitly giving discrete change points and interval labels may sound reasonable statistically but provides limited interpretability. For example, Turkcan et al.^150^ developed a Bayesian inference scheme applicable to confined receptor trajectories that can reconstruct local diffusivity and a confining potential and reported a force map spanning 0–0.28 pN for CPεT-receptor tracks and noted that D can be inferred locally via sliding time windows. Without explicit segments, however, no dwell boundaries can be inferred with confidence, and we cannot characterize the diffusion state inside a trajectory, limiting the biological interpretability of the method. Therefore, robust segmentation, which provides time-stamped state assignments to the whole trajectory, is preferred to describe intratrajectory diffusion heterogeneity. It is noticeable that there are two types of segmentation. One type of method provides continuous state labels or parameter estimations (e.g., D­(t) or α­(t)) to each frame or fixed-length interval. The other type of method segments a trajectory into discrete, varying length intervals corresponding to different states.

There have been several non-ML strategies developed to achieve segmentation. Godoy et al.^151,152^ estimate time-varying parameters using a sliding-window EM approach and then define boundaries through change-detection methods, specifically likelihood-ratio or Kullback–Leibler statistics. They emphasize that the choice of window length directly influences sensitivity, as overly long windows can smear short dwells, while overly short windows may lead to oversegmentation of noise. ExTrack^120^ fits a multistate HMM to recover diffusion, localization error, and transition rates and assigns per-frame state probabilities; for tractability on short/noisy tracks, it replaces long HMMs with a 3–7 frame window approximation, trading some accuracy for speed. As a window-free alternative, Lanoiselée et al.^153^ use a recurrence-matrix computed from all pairwise distances; square blocks along the matrix diagonal mark trapped segments, enabling transient-trapping detection without preset windows. These non-ML methods have limitations. Windowed EM+CD and HMMs require model choices (state count, Markovianity, noise handling) and can be window-sensitive, while recurrence-matrix analysis avoids window bias yet is presently tailored to a small set of scenarios involving trapping motifs.

Recent ML/DL approaches have made notable contributions in addressing limitations originating from a lack of segmentation methods or poor segmentation of conventional methods. These methods can be classified into supervised methods that assume a fixed set of interpretable states and learn to detect transitions between them on single-particle tracks and nonsupervised methods that do not assume particular diffusion models and instead learn latent representations of motion from the trajectories themselves. Among methods aiming to segmenting whole trajectories into predefined diffusion states, vbSPT remains a popular parametric method and, in head-to-head tests on thousands of simulated trajectories, exhibits low bias in diffusion and bound-fraction estimates compared with single-trajectory MSD fitting (bias ∼0.8% and 5% vs ∼39.6% and 22%, respectively).^146^ Building on this foundation, DeepSPT^42^ uses an ensemble of fully convolutional networks to classify each time point of a trajectory as normal, directed, confined, or subdiffusive. The segmentation module ingests *x*/*y*(/z) coordinates and outputs per-frame probabilities; it was trained on 900,000 simulated trajectories spanning 4 orders of magnitude in diffusivity and varying localization errors. Evaluation on 20,000 heterogeneous test trajectories showed a median per-trace accuracy of 96% and an F1-score of 0.88, with subdiffusive, normal, and confined motions correctly labeled 86–96% of the time. U-AnDi^155^ uses a U-Net architecture with dilated causal convolutions and gated activation units to perform semantic segmentation of anomalous diffusion; it detects changes in the anomalous exponent and dynamic model, consistently outperforming other methods across segmentation tasks and aligning closely with experimental data on membrane protein diffusion (Figure 12b). Hybrid sequence-to-sequence RNN models have also been proposed. Martinez et al.^156^ reported an LSTM network that was trained from synthetic trajectories that switch between anomalous and normal diffusion and reported ∼95% classification accuracy on a test set. They noted that smoothing predictions with a moving window improves interpretability but may overpredict change points. DL-MSS^102^ is another example of a hybrid method that also uses an LSTM to segment trajectories into short “tracklets” of uniform mobility and then clusters these tracklets in moment scaling space to recover the number of mobility classes and their physical parameters; the network achieves ∼92% accuracy on test data.

Complementing these supervised tools are unsupervised and self-supervised approaches that learn latent representations of motion without prespecifying diffusion categories. We have extensively discussed Deep-SEES^105^ and NOBIAS^103^ before. Deep-SEES embeds sliding subtrajectories with a variational LSTM autoencoder and clusters the latent vectors to segment tracks without predefining state number or type, whereas NOBIAS couples a nonparametric HDP-HMM to infer the unknown state sequence with a pretrained RNN that assigns each inferred state to a physical diffusion model (e.g., Brownian, FBM, CTRW, Lévy), yielding data-driven segmentation with interpretable posthoc semantics. The RANDI method^97^ similarly employs recurrent networks to infer the anomalous exponent, identify the diffusion model, and segment trajectories switching between behaviors and ranked among the top three methods across all tasks in the anomalous diffusion challenge at the time of publishing (Figure 12c).

ML/DL methods have contributed significantly to addressing previously discussed issues in non-ML methods. Supervised ML/DL segmenters such as DeepSPT,^42^ U-AnDi,^155^ and DL-MSS^102^ replaced fixed-length sliding windows by learned temporal context (CNN/LSTM/attention), yielding per-frame categorical probabilities, explicit change points, and dwell-wise parameters that remain robust under low SNR, motion blur, and variable dwell lengths. In doing so, they reduced reliance on hand-tuned model choices that burden non-ML HMM/EM pipelines (state count, Markovianity, and stationary noise) and generalized beyond trapping motifs to multistate mixtures within single trajectories. Unsupervised/self-supervised methods such as Deep-SEES,^105^ NOBIAS,^103^, and RANDI^97^ further made it independent of a priori assumptions by discovering state number/structure in learned latent spaces (or via HDP-HMM), mitigating window bias and model misspecification while allowing posthoc mapping to physical diffusion modes.

Deep learning methods have already delivered various new biological insights that were inaccessible to classical analyses. Methods such as DeepSPT, DL-MSS, Deep-SEES, and NOBIAS have already revealed organelle-specific diffusion signatures, viral entry states, and substrate-binding alternations, underscoring the biological relevance of ML-based analyses.^102,103,105,154^

Apart from 1D intensity trace or trajectories from SPT, there are also ML/DL based methods that use different forms of input data. Bound2Learn^53^ is an RF classifier trained on physical descriptors of single-molecule tracks output from TrackIt to distinguish DNA-bound proteins from freely diffusing ones. When tested on a simulated *Escherichia coli* timelapse with a ground truth 8 s bound time, Bound2Learn recovered an ∼7 s mean residence and achieved 0.93 accuracy. By contrast, an MSD heuristic on the same data achieved only 0.54 accuracy and produced a bound-time estimate of much lower than 8 s. Mitometer^157^ is a context-specific pipeline that uses morphological and intensity features from super-resolution microscopic images to segment and track individual mitochondria and then uses an RF classifier to distinguish mitochondrial phenotypes in triple-negative breast cancer cells versus receptor-positive counterparts. When benchmarked on standard segmentation metrics, the threshold-based Mitometer achieved a Dice coefficient of 0.76, a mean Intersection-over-Union of 0.63, and a pixel-accuracy of 0.84. MoDL^158^ is a deep learning method used for mitochondria segmentation that achieves remarkably higher segmentation accuracy (Dice 0.92, mIoU 0.84, and PA 0.95) compared to Mitometer. Trained on approximately 20,000 manually annotated SR images for contour learning and an extended data set of more than 100,000 images linked to biochemical assays, MoDL integrates high-fidelity masks with functional predictions (MMP, respiration, ROS, ATP, mitophagy). It maintains strong performance across diverse cell lines and imaging platforms, making it a robust image-level solution when both high-quality segmentation and reliable downstream functional readouts are required.

## 7 Current Limitations and Challenges

Despite the successes of ML/DL in the SPT, several challenges limit their broader application. A primary challenge is the lack of generalized training data sets. Deep learning models typically require large, high-quality labeled data sets, yet obtaining extensive annotated SPT data is difficult in practice. Therefore, the simulated trajectories or small experimental data sets were used for training, which can lead to overfitting and poor domain generalization. Models that perform well under specific training conditions can fail when applied to different imaging setups, noise levels, or particle types not seen during training. The sensitivity to distribution shifts (out-of-distribution data) means that a network might misbehave or degrade significantly outside its narrow training domain. In essence, without access to abundant and diverse training data, deep learning models are prone to learning misleading patterns that are specific to the training set, which limits their ability to generalize reliably to new experimental conditions.

Another commonly discussed challenge is the “black box” nature (lack of interpretability) of deep learning models. Unlike traditional analytical methods or simpler statistical models that yield physically meaningful parameters, the internal reasoning of a deep neural network is largely opaque. Biophysicists and microscopists may be uneasy, trusting a result that cannot be easily explained in terms of known physics or intuitive features. This lack of transparency makes it difficult to extract meaningful biophysical insights from a trained model’s decisions beyond simply accepting the model’s output. For example, understanding why a particular trajectory was labeled as “confined” rather than “diffusive.” The lack of explainability can reduce user confidence and hinder adoption of the field that values mechanistic understanding. While techniques such as feature attribution and hybrid modeling are being developed to address this issue, creating truly interpretable deep learning models for single-particle tracking remains an unsolved challenge.

Quantifying uncertainty in deep learning-based SPT analysis is another significant challenge. Traditional methods, such as Bayesian state models, inherently provide confidence intervals or probability estimates. In contrast, most deep neural networks produce only point predictions, offering little to no information about how confident the model is in its output. Conventional deep learning classifiers often exhibit overconfidence in their predictions, meaning that a softmax score or classification label should not be taken at face value as a well-calibrated probability. Although methods for quantifying and reporting uncertainty in deep networks are actively being developed, their integration into SPT workflows remains a challenge. This lack of uncertainty output, combined with limited interpretability, can further reduce trust in automated results.

Moreover, there is a disconnect between purely data-driven DL models and physics-informed modeling in SPT. Single-particle motion is governed by physical principles (e.g., diffusion laws, force-field interactions, confinement by cellular structures), but most deep learning approaches do not inherently enforce or incorporate such prior knowledge. As general function approximators, current models can sometimes learn about unphysical relationships or overlook known constraints. Bridging this gap by integrating physical models or constraints into deep learning remains an active and evolving area of research. So far, ML/DL to SPT has treated the data in a model-agnostic way, which can lead to predictions that are difficult to align with established biophysical principles or that break down when applied outside the training conditions. Bridging the gap between data-driven algorithms and theoretical models is essential for generating results that are both accurate and physically meaningful.

Finally, computational limitations remain a significant practical challenge. Advanced deep learning models used for particle localization, tracking, or classification typically require extensive computational resources and long training times. In experimental contexts where real-time or online analysis is needed (e.g., closed-loop particle tracking), the latency and hardware requirements can be a problem. To make deep learning more practical for SPT, especially at scale, there is a strong need for streamlined models, efficient algorithms, and accessible hardware solutions.

In summary, while deep learning has opened exciting opportunities for single-particle tracking, it also introduces unique challenges: the need for large labeled data sets, risk of overfitting, limited generalization, lack of interpretability, absence of uncertainty estimates, difficulty integrating physical principles, and high computational costs. Overcoming these obstacles is crucial for building trust and realizing the full potential of deep learning in SPT. Ongoing efforts are addressing these gaps by improving model robustness and interpretability, developing uncertainty quantification techniques, curating benchmark data sets, and exploring hybrid approaches that combine data-driven learning with physical modeling. These will help advance the field and enable broader adoption in quantitative applications.

## 8 Outlook: Opportunities and Future Directions

The integration of deep learning into SPT promises transformative developments in several key directions. Real-time adaptive imaging is an emerging frontier, where AI-driven microscopes dynamically adjust imaging parameters in response to observed particle behavior, optimizing data collection and enabling the capture of transient molecular events.^37,159^ Coupled with automated experimental design, these intelligent systems could autonomously identify and pursue informative data, significantly enhancing experimental efficiency.^160^ Advances in next-generation neural architectures, such as transformer models and graph neural networks, offer improved capabilities to capture complex temporal dynamics and interparticle relationships inherent in SPT data.^161^ Concurrently, novel training paradigms like federated learning and simulation-to-reality transfer are poised to address data scarcity, improve generalization, and facilitate community-wide model sharing without compromising data privacy.^162^

Longer-term, significant potential lies in developing physics-informed and -interpretable AI tools tailored specifically for molecular tracking. Integrating known physical laws directly into AI models can ensure physically consistent and more reliable predictions.^163^ Simultaneously, a focus on transparency through explainable AI methods will help demystify model predictions, enabling researchers to gain deeper insights into particle dynamics. Ultimately, these developments indicate a future where AI serves not merely as a computational tool but as an integrated partner in single-particle tracking research, driving discoveries with precision, reliability, and clarity.

In summary, machine learning is already reshaping single-particle tracking by expanding the possibilities for data analysis and interpretation. Integrating machine learning with single-particle tracking is accelerating discoveries in molecular dynamics from basic biophysics to biomedical applications. As these techniques continue to evolve, single-particle studies are becoming more quantitative, comprehensive, and insightful. The future of SPT will be defined by the convergence of advanced imaging modalities, intelligent data analysis, and a holistic understanding of molecular motions that orchestrate biological life.

## Abbreviations

SPT: single-particle tracking
ML: machine learning
DL: deep learning
MSD: mean square displacement analysis
HMMs: hidden Markov models
2D-SPT: two-dimensional single-particle tracking
3D-SPT: three-dimensional single-particle tracking
EFM: epifluorescence microscopy
TIRFM: total internal reflection fluorescence microscopy
HILO: highly inclined and laminated optical sheet
LSFM: light sheet fluorescence microscopy
SNR: signal-to-noise ratio
PSF: point spread function
DH-PSF: double-helix PSF
SLMs: spatial light modulators
MPM: multifocal plane microscopy
SPADs: single-photon avalanche diodes
3D-PART: 3D position acquisition via rapid tracking
2D-EOD: two-dimensional electro-optical deflector
TAG: tunable acoustic gradient
APD: avalanche photodiodes
DNNs: deep neural networks
CNNs: convolutional neural networks
FCNs: fully convolutional networks
RNNs: recurrent neural networks
LSTM: long short-term memory
GPU: graphic processing unit
TPUs: tensor processing units
FPGAs: field-programmable gate arrays
FBM: fractional Brownian motion
CTRW: continuous-time random walk
LW: Lévy Walks
TAMSD: time-averaged mean squared displacement
VACF: velocity autocorrelation function
PSD: power spectral density
GRUs: gated recurrent units
VB-HMM: variational Bayesian HMM
iHMM: infinite HMM
GMMs: Gaussian mixture models
ANDI: anomalous diffusion
MSS: moment scaling spectrum
CLDNN: convolutional/LSTM deep neural network

## Vocabulary Section

Resolution: The smallest distance at which two objects can be distinguished as separate. In optical microscopy, it is limited by diffraction and depends on wavelength and numerical aperture.
Precision: The reproducibility of repeated measurements under identical conditions. In optical imaging, it quantifies the consistency of localization or intensity estimates, independent of their proximity to the true value.
Signal-to-noise ratio (SNR): The ratio of the desired signal to background noise, serving as a key indicator of image quality. A higher SNR facilitates more reliable detection and improves localization accuracy.
Diffraction-limited spots: Due to the wave nature of light, a point source is imaged not as a perfect point but as a diffraction pattern defined by the point spread function. Such “diffraction-limited spots” typically have sizes of a few hundred nanometers in visible light microscopy.
Subpixel accuracy: Subpixel accuracy describes the ability to localize features with precision finer than the detector’s pixel size, typically achieved by fitting the point spread function. This is essential for single-molecule localization microscopy.
Uncertainty quantification: Uncertainty quantification involves the systematic estimation of confidence intervals or error bounds in measurements. In optical imaging, it provides a statistical framework to assess the reliability of localization results and model predictions.
